# Supplementary material for: Large-scale phylogenomics of aquatic bacteria reveal molecular mechanisms for adaptation to salinity
Source: Sci Adv. 2023 May 26;9(21):eadg2059. doi: 10.1126/sciadv.adg2059 (PMC10219603; doi:10.1126/sciadv.adg2059)
Supplement: 20230526-1 [file sciadv.adg2059.v1.pdf]

MICROBIOLOGY

Large-scale phylogenomics of aquatic bacteria reveal molecular mechanisms for adaptation to salinity

Krzysztof T. Jurdzinski[1]†, Maliheh Mehrshad[2]†, Luis Fernando Delgado[1], Ziling Deng[1], Stefan Bertilsson[2], Anders F. Andersson[1]*

Copyright © 2023 The Authors, some rights reserved; exclusive licensee American Association for the Advancement of Science. No claim to original U.S. Government Works. Distributed under a Creative Commons Attribution License 4.0 (CC BY).

The crossing of environmental barriers poses major adaptive challenges. Rareness of freshwater-marine transitions separates the bacterial communities, but how these are related to brackish counterparts remains elusive, as do the molecular adaptations facilitating cross-biome transitions. We conducted large-scale phylogenomic analysis of freshwater, brackish, and marine quality-filtered metagenome-assembled genomes (11,248). Average nucleotide identity analyses showed that bacterial species rarely existed in multiple biomes. In contrast, distinct brackish basins cohosted numerous species, but their intraspecific population structures displayed clear signs of geographic separation. We further identified the most recent cross-biome transitions, which were rare, ancient, and most commonly directed toward the brackish biome. Transitions were accompanied by systematic changes in amino acid composition and isoelectric point distributions of inferred proteomes, which evolved over millions of years, as well as convergent gains or losses of specific gene functions. Therefore, adaptive challenges entailing proteome reorganization and specific changes in gene content constrains the cross-biome transitions, resulting in species-level separation between aquatic biomes.

INTRODUCTION

Transitions between different environmental conditions have long been identified as one of the major drivers of evolutionary innovation (*1*). Evidence of fast dispersal of bacteria across the globe suggests that physicochemical and biological factors constrain the distribution of bacteria to a higher extent than geography and dispersal limitation (*2*). However, the highly dynamic nature of bacterial genomes (*3*), including the ability to acquire new genes through horizontal gene transfer (*4*), can potentially facilitate the crossing of environmental barriers.

Salinity, pH, temperature, and other factors important for structuring microbial diversity are predicted to change in coastal waters because of climate change (*5*). Combined with the crucial role of bacteria in such ecosystems (*6*), there is a need to understand the potential and limitations of adaptation and dispersal in response to changing environmental conditions. Comparative genome analyses across major environmental barriers have the potential to uncover the molecular mechanisms of such adaptation. Conducting the analysis in a phylogenetic framework allows for identifying genetic traits that have been convergently gained or lost in multiple lineages in conjunction with switching from one environment to another. Such traits are likely to be adaptive, in contrast to many other traits that may differ in prevalence between the biomes because of different taxonomic composition, i.e., through "hitchhiking" in genomes where other traits have been selected for by the environment.

Salinity has long been considered the strongest physicochemical factor determining bacterial community composition (*7*, *8*). Distinct bacterial lineages inhabit freshwater and marine biomes, and transitions between these biomes have been rare in the documented evolutionary history of aquatic bacteria (*9*–*11*), pinpointing differences in salinity as a particularly strong barrier for microbes to overcome. More recently, fragment recruitment–based metagenomic analyses have indicated that brackish environments with intermediate salinity levels [between 0.5 and 30 Practical Salinity Units (PSU)] also host genetically distinct bacterial lineages (*12*, *13*). However, this has not been confirmed in a rigorous phylogenomic framework, and little is known about the biogeography of the potential brackish microbiome and its relationship with its freshwater and marine counterparts. Key questions remain unanswered, such as which groups of bacteria have transitioned between the aquatic biomes, how often, in which directions, and when have the transitions taken place.

Protein properties such as isoelectric point distributions were recently shown to differ between aquatic biomes with a pattern that may reflect adaptation to different extra- and intracellular ion concentrations (*14*). Detected changes are mainly seen for surfaces of soluble proteins and differ between certain groups of closely related freshwater, brackish, and marine lineages (*14*). Likewise, the functional gene content of microbial communities changes in a salinity-dependent manner (*11*, *12*, *15*–*17*), although it is unclear to what extent these effects are merely attributable to differences in the taxonomic composition. Widespread patterns of convergent gene gain and loss accompanying cross-biome transitions, as well as evolutionary factors and dynamics driving the shift in isoelectric point distribution, could be further elucidated through a systematic phylogenomic analysis across the bacterial tree of life.

Here, we analyze an extensive and diverse set of freshwater, brackish, and marine metagenome-assembled genomes (MAGs) in a phylogenomic framework. We cluster MAGs by average nucleotide identity (ANI) and compare the similarities of genomes from the same and different biomes. We identify the most recent transitions between the aquatic biomes and estimate their evolutionary timeline and directionality. Last, we show that large-scale changes in the properties of the predicted proteome and specific alterations

[1]Department of Gene Technology, KTH Royal Institute of Technology, Science for Life Laboratory, Stockholm, Sweden. [2]Department of Aquatic Sciences and Assessment, Swedish University of Agricultural Sciences, Uppsala, Sweden.
*Corresponding author. Email: anders.andersson@scilifelab.se
†These authors contributed equally to this work.

in gene content accompany cross-biome transitions in diverse bacterial lineages.

RESULTS

A comprehensive dataset of MAGs from freshwater, brackish, and marine biomes was compiled

A total of 13,783 MAGs reconstructed from freshwater (*18*–*20*), brackish (*17*, *21*), and marine (*22*, *23*) biome metagenomic datasets (Fig. 1A) were downloaded to analyze evolutionary histories and functional adaptations in aquatic bacteria. These datasets come from studies designed to cover a wide range of niches mostly within the epipelagic (sunlit) zone via sampling at different depths and across environmental gradients. The MAGs were filtered at ≥75% completeness and ≤5% contamination thresholds, and the 11,509 MAGs passing these criteria were considered for the remaining analyses (11,248 bacterial and 233 archaeal). For further analyses, we focused on the bacterial MAGs, of which 7643 were from fresh water, 2240 were from brackish water, and 1365 were from marine waters (Table 1). Collected MAGs contained representatives from 72 phyla distributed across 135 classes and 348 orders, with the majority (91%) belonging to only 12 bacterial phyla with >100 representatives per phylum (data S1).

Bacterial species are rarely shared between biomes

Clustering the bacterial MAGs using ANI at the threshold of the operational definition of species [95% (*24*, *25*)] resulted in 3561 genome clusters (Fig 1B). MAGs of the vast majority of genome clusters (n = 3547) belonged to a single biome (2063 freshwater, 485 brackish, and 999 marine genome clusters), while no genome cluster had representatives from all three biomes. Only 14 genome clusters harbored MAGs belonging to pairs of biomes [nine, four, and one genome clusters with freshwater-brackish (FB), brackish-marine (BM), and freshwater-marine (FM) MAGs, respectively; Fig 1C]. The only shared FM genome cluster was affiliated to the genus *Limnobacter* (phylum Proteobacteria). Shared FB clusters were affiliated to phyla Actinobacteriota, Proteobacteria, Verrucomicrobiota, and Bacteroidota. The shared BM clusters belonged to phyla Proteobacteria, Verrucomicrobiota, and Bacteroidota.

Forty-five of the 485 brackish genome clusters had members from both included brackish water bodies, the Baltic Sea and the Caspian Sea, while 300 and 141 were unique to Baltic and Caspian, respectively. The shared clusters belonged to 13 classes in 10 different phyla (Planctomycetota, Verrucomicrobiota, Gemmatimonadota, Bacteroidota, Actinobacteriota, Chloroflexota, Cyanobacteria, Firmicutes, Nitrospinota, and Proteobacteria). The extent of overlap between Baltic Sea and Caspian Sea genome clusters (18.6%) was significantly higher than between brackish and freshwater (0.7%) and brackish and marine clusters (0.5%; $P < 10^{-16}$ for both comparisons), despite the geographic separation and lack of hydrological connectivity between these ecosystems. This supports the previously postulated existence of a global brackish-specific microbiome (*12*, *21*).

The clustering results above indicate that bacterioplankton belonging to the same species (within the 95% ANI threshold) are inhabiting the geographically distinct and distant Baltic and Caspian seas. To investigate intraspecific (within-species) biogeographical patterns, we used a population genomics approach, where allele frequencies in single-nucleotide variant (SNV) loci are obtained by aligning metagenomic sequences to the genomes. As previously reported (*26*), principal coordinate (PCoA) analysis of pairwise fixation indices (F_{ST}) showed that for each of the five genome clusters where we applied this approach in the present study, populations were highly structured according to salinity within the Baltic Sea (Fig. 2A). However, adding the Caspian Sea data to the analysis revealed even stronger population structuring between the two seas (Fig. 2B), indicating geographic separation at the strain level within these species.

Transitions between aquatic biomes are rare, ancient, and, in most cases, directed into the brackish biome

We reconstructed the phylogeny of the aquatic MAGs using a set of conserved housekeeping genes (*27*). The resulting tree was pruned to contain a maximum of one MAG from each biome per previously identified genome cluster (i.e., ~species; Fig. 3). The most recent transitions between the aquatic biomes, if detected, should be represented on the phylogenetic tree as pairs of closest related monophyletic and biome-specific groups. We call these clades monobiomic sister groups (MSGs; Fig. 4A). We identified 310 MSG pairs, with brackish and marine (BM; n = 136) and freshwater and brackish (FB; n = 119) transitions being twice as numerous as freshwater and marine (FM; n = 55), despite the larger number of freshwater and marine MAGs in the pruned tree (Fig. 4B). The number of FB and FM transitions was also significantly lower than would be expected if phylogeny would not correlate with

Table 1. Number of MAGs originated from each dataset.

Dataset	#Metagenomes	#Total MAGs	#Filtered MAGs	#Filtered Bacteria	#Filtered Archaea	Reference
North American lakes (F)	141	193	84	84	0	(*18*)
StratFreshDB (F)	267	8554	7654	7554	100	(*20*)
Lake Baikal (F)	2	35	7	5	2	(*19*)
Baltic Sea (B)	123	1989	1989	1954	35	(*17*)
Caspian Sea (B)	3	324	324	314	10	(*21*)
Global Ocean T (M)	243*	2631	990	932	58	(*22*)
Global Ocean D (M)	243*	957	461	433	28	(*23*)

*The same metagenomes were used to obtain MAGs in (*22*, *23*) but using different approaches.

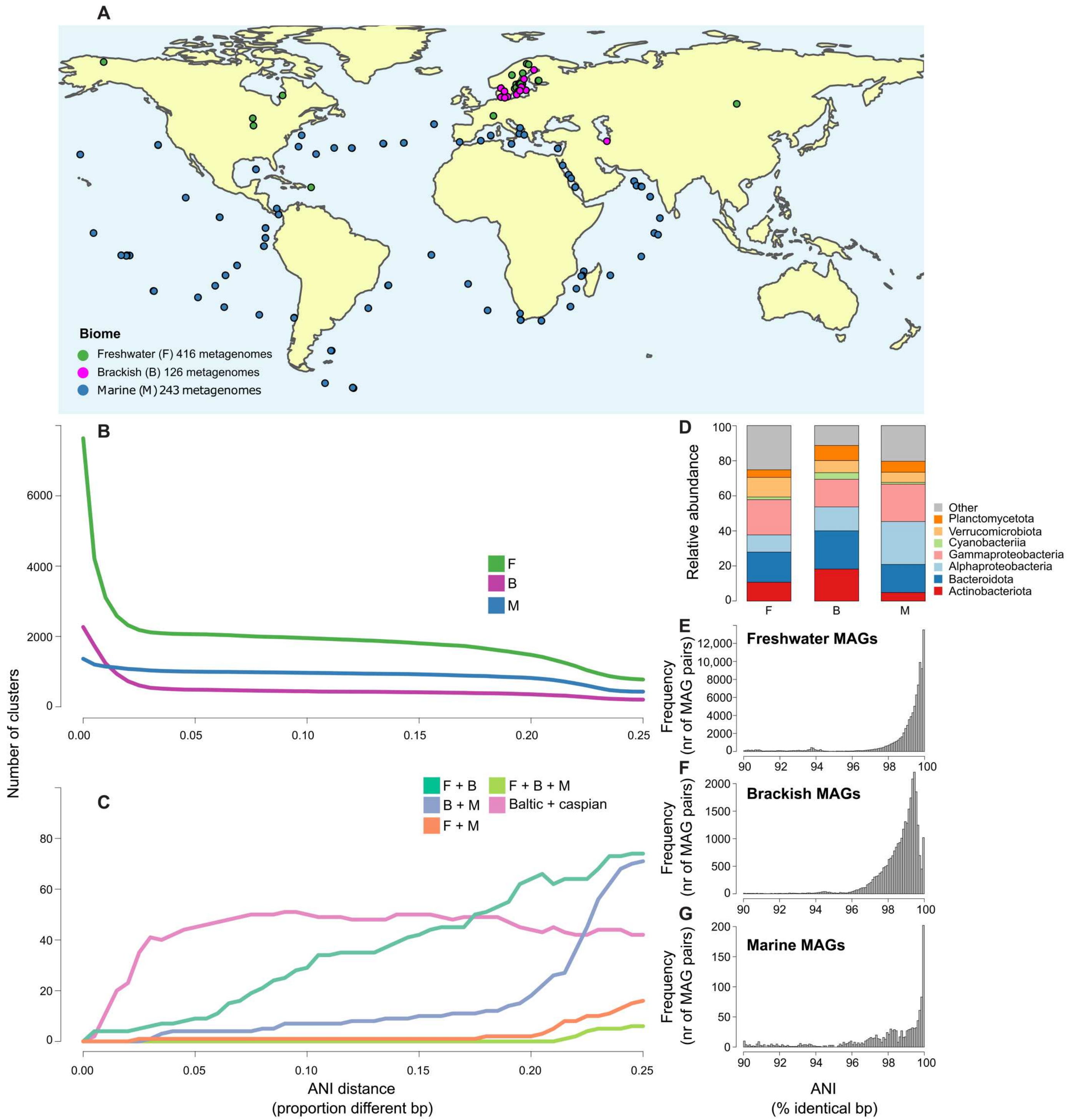

Fig. 1. Status of shared bacterial clusters in different biomes. (**A**) Map showing origins of MAGs (*17–23*). (**B**) Number (nr) of MAG clusters at different nucleotide distance cutoffs in each biome. (**C**) Number of MAG clusters at different nucleotide distance cutoffs with members of different biome combinations. (**D**) Relative abundance of MAGs affiliated to different phyla in the three different biomes. Frequency distributions of pairwise inter-MAG ANI values in the range of 90 to 100% in (**E**) freshwater, (**F**) brackish, and (**G**) marine genomes. bp, base pairs.

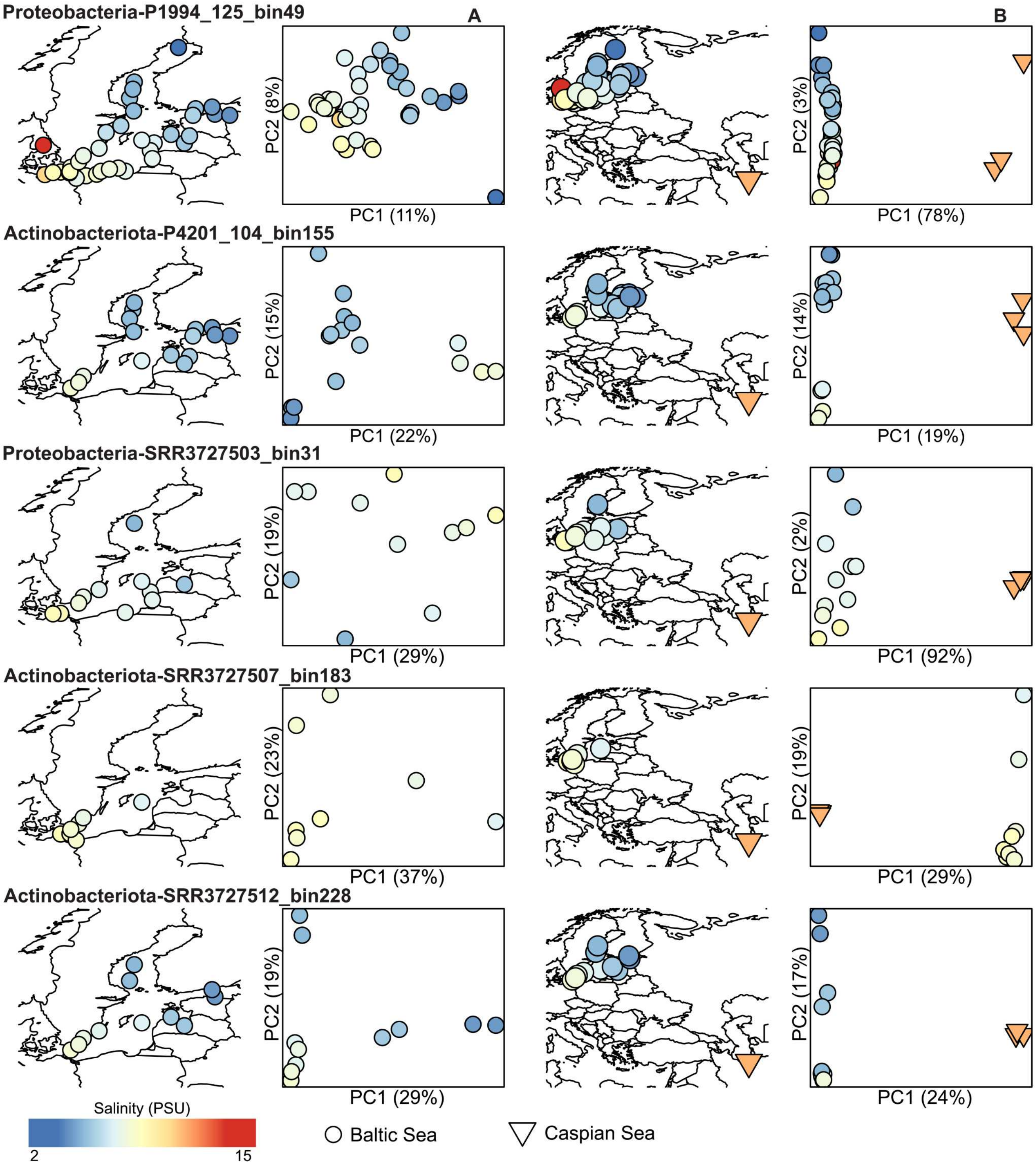

Fig. 2. Population structure of brackish genome clusters. (**A** and **B**) Left: Geographic origin of each metagenome sample included for the MAG. Right: A PCoA based on pairwise F_{ST} values. Only Baltic Sea samples were included in (A), whereas both Baltic Sea and Caspian Sea samples were included in (B).

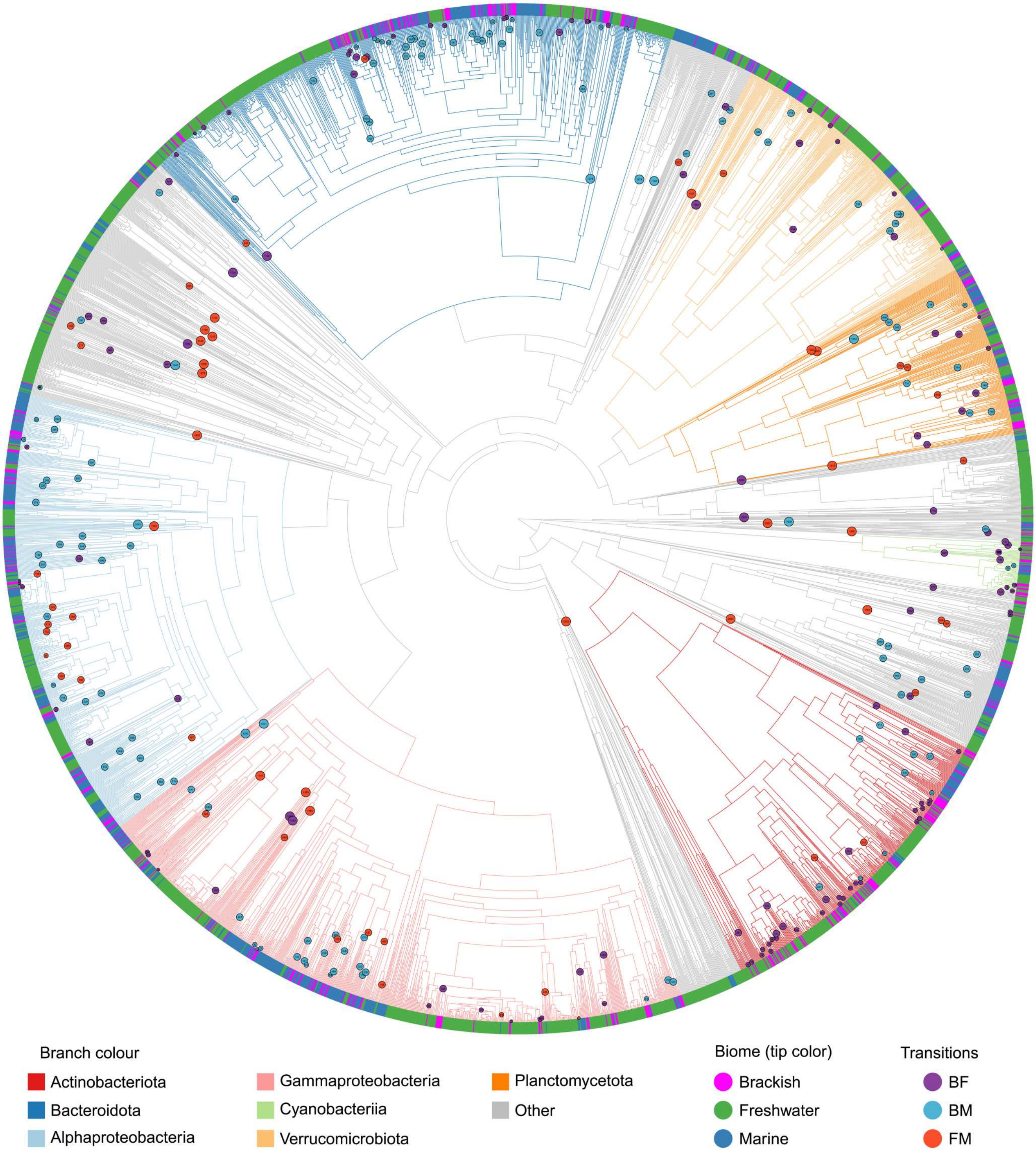

Fig. 3. Reconstructed phylogeny of MAGs of the three aquatic biomes. Tree with branch lengths corresponding to estimated minimal times since divergence. Only tips present in the pruned tree are visualized. Identified transitions, i.e., nodes corresponding to the most recent common ancestors (MRCAs) of MSG pairs, are color-labeled by transition type.

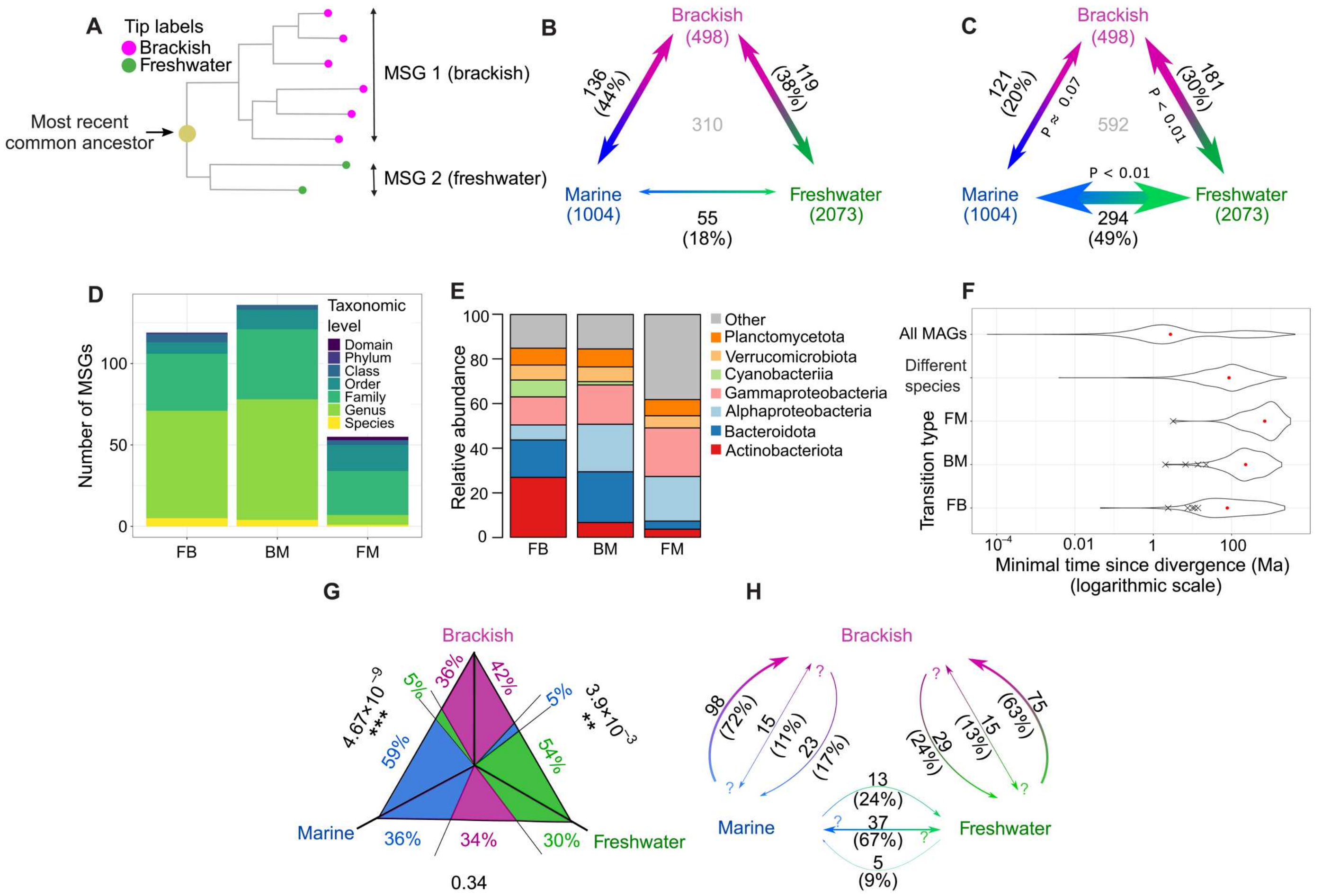

Fig. 4. The most recent transitions between biomes. (**A**) Illustration of an MSG pair corresponding to an FB transition. (**B**) Observed numbers of transitions between the biomes. Numbers of MAGs included in the pruned tree are given in brackets under the biome names. The total number of all identified cross-biome transitions is shown in gray in the middle of the plot. (**C**) Expected numbers of transitions between the biomes as predicted by random permutations of biome annotations. On the internal sides of the arrows are *P* values for the estimated numbers being smaller (for FM and FB) or bigger (for BM) than the observed numbers of transitions. (**D**) The number of transitions distributed across the lowest shared taxonomic level to which both MSGs could be classified. If no annotation was available at a taxonomic level, then a higher one was considered. (**E**) Proportion of pairs of MSGs for broader taxonomic groups. (**F**) The distributions of estimated minimal times since divergence for all nodes on the unpruned tree ("All MAGs"), nodes corresponding to most recent divergence events [pairs of bacterial species sharing an exclusive MRCA on the pruned tree, i.e., "Different species"], and nodes corresponding to transitions grouped by type. The timing of transitions within >95% ANI clusters is marked as "X." (**G**) Mean likelihoods of ancestral biome states for transition between the biomes. On the sides of the triangle *P* values are given for difference in the likelihood for ancestral states of the two biomes in which the MSGs for the transition type were identified (for example, between brackish water and fresh water for FB). (**H**) Numbers (and proportion) of transitions of each type for which the direction of the transition indicated by the arrows was more probable than the two other ancestral biome states taken together. Both-sided arrows indicated transitions with no identified dominant transition direction.

biome annotation (Fig. 4C). This corroborates observations for the previously described genome clustering.

Most of the identified FB and BM MSG pairs belonged to the same genus (around 55% in both cases), while for FM transitions, the lowest-level shared taxonomic annotation was most often (49%) at the family level (Fig. 4D and table S1). Only small proportions (1.8 to 4.2%) of the MSG pairs belonged to the same species [defined either by Genome Taxonomy Database (GTDB) annotation or as a >95% ANI genome cluster]. The taxonomic distribution of MSG pairs followed the overall distribution in the corresponding biomes for BM and FM, but not for FB, probably because of the overrepresentation of Actinobacteriota and Cyanobacteria in this transition type (Fig. 4E and fig. S1).

We estimated the minimal time since divergence of the MSG pairs on the basis of phylogenetic distances using a method that allows for variable evolutionary rates (Figs. 3 and 4F and fig. S2) (*28*). As time constraints, we used minimal times since divergence of genomes of endosymbiotic bacteria, known from fossil records of their hosts (table S2) (*29*–*37*). As obligate endosymbionts have high evolutionary rates that exceed those of free-living bacteria (*37*), this model gives conservative estimates of time since the transitions started. The timing differed depending on the transition type ($P < 10^{-6}$ for all comparisons; Fig. 4F), FB being the most recent and FM being the oldest. The difference in median times since FB and BM transitions could not be attributed to the uneven representation of biomes in the dataset (fig. S2B).

The most recent transition was estimated to have happened at least 44.8 thousand years (ka) ago, and all other transitions were millions of years ago (table S3). Therefore, all the detected transitions started long before the current brackish conditions were established in the Baltic Sea [8 ka ago (*38*)] and only the six most recent ones to the period since the Caspian has continuously been brackish [2 to 3 million years (Ma) ago (*39*)]. These results imply that the evolutionary history of the global brackish microbiome (*12*) by far predates the formation of these two brackish basins.

To infer the directions of the biome transitions, we conducted ancestral (biome) state reconstructions of the most recent common ancestors (MRCAs) of the MSG pairs, correcting for the uneven representation of biomes in the pruned tree (see Materials and Methods). FB and BM transitions were generally more likely to happen into than out of the brackish biome, while for FM transitions, no significant direction preference was observed (Fig. 4, G and H, and figs. S3 and 4).

Large-scale changes in proteome properties follow cross-biome transitions

We next assessed if changes in proteome properties accompany the biome transitions. The average distributions of isoelectric points (pIs) of the proteins encoded in the genomes were compared between MSGs in a pairwise manner. Significant differences ($P < 10^{-5}$) in the proportion of neutral (pI $\in$ [5.5, 8.5)) proteins were observed for all the transition types (Fig. 5, A and B, and data S3), with a higher proportion in the biomes of lower salinity. The frequencies of acidic proteins (pI $\in$ [3.0, 5.5)) showed the opposite pattern and were significantly different ($P < 10^{-5}$) for the comparisons that included freshwater MSGs but not for the BM transition type. The results suggest that the higher representation of neutral pIs observed in freshwater bacteria (*14*) gradually diminishes with increasing salinity, while low proportion of acidic proteins is a distinctive feature of freshwater bacteria. However, the extent of transition-related changes in pI distribution highly depended on taxonomy, with particularly small changes for Actinobacteria (fig. S5).

Polar, charged, and acidic amino acid frequencies broadly increased ($P < 10^{-5}$) with salinity across the MSG pairs (Fig 5C and data S3). Changes across all the transition types were significant ($P < 10^{-6}$) for acidic amino acids, which is a subcategory of the other two categories and may thus also explain the changes observed for those. In contrast, the frequency of basic amino acids differed significantly ($P < 10^{-5}$) only for BM transitions.

Among the acidic amino acids, there was significantly more glutamate in proteomes inferred from relatives living in higher-salinity biomes across all the transition types ($P < 0.01$), and the difference between the biomes was bigger for glutamate than for aspartate in all the cases (fig. S6 and data S3). For BM transitions, the higher proportion of basic residues in marine proteomes could be attributed to lysine. Big and significant differences ($P < 0.001$) in the frequency of alanine across BM and FM transitions suggest that this nonpolar amino acid with a relatively "neutral" effect on protein structure (*40*) is often substituted by charged residues in transitions to higher salinity. For all transition types, there was significantly less ($P < 0.001$) proline in the predicted proteomes at higher salinity.

To investigate the dynamics of how the proteome is reshaped after biome transitions, we compared the differences in pI distributions to inferred times since divergence. The overall change in pI distributions followed different logarithmic relationships for within-MSG (no transition) divergence times as compared to any of the transition types (Fig. 5D). Moreover, the differences in pI distribution across transitions were consistently larger than changes resulting from within-MSG (no transition) divergence events when the latter were at least 44 to 181 million years more recent (Fig. 5, G to I). This suggests that proteomes are under strong selection and continue to evolve to match the new salinity conditions long after the switch to a new biome (fig. S7).

Specific changes in gene content accompany cross-biome transitions

For each transition type, we were able to identify specific functional genes [Kyoto Encyclopedia of Genes and Genomes (KEGG) orthology groups (KOs)], significantly overrepresented [false discovery rate (FDR)–corrected $P < 0.1$] in the MSGs of one of the compared biomes after correcting for differences in estimated completeness of the MAGs (Fig. 6 and data S4). However, transitions did not change the gain and loss rates (Fig 5E), although gene numbers significantly differed for BM transitions (Fig. 5F and data S3), with brackish MAGs having a median of 386 more genes. Therefore, the gains or losses of the same gene functions (KOs) that we observed in multiple independent transition events most likely result from analogous changes in selective pressures caused by the biome transitions.

Transporters were the only functional category (KO level C) overrepresented (FDR-corrected $P < 0.05$) in all transition types (Fig. 6 and fig. S8), some with known roles in adaptation to altered salinity. For FB transitions, brackish MAGs more frequently contained components of two [or of the same (*41*)] potassium uptake systems (K03498 and K03499) co-acting to allow growth in higher osmotic pressure (*42*). A component of the uptake system with higher affinity, K03499 (*42*), was also more prevalent in marine genomes across FM transitions. In addition, for both FB and FM MSG pairs, a Na^+/Ca^{2+} antiporter (K07301) was more often found in the more saline biomes. Analogous changes in presence of the same K^+ uptake systems and of Na^+/Ca^{2+} antiporters were reported in a comparison of terrestrial and marine Flavobacteriaceae (*43*). For BM MSG pairs, adenosine triphosphate–dependent phosphate transport genes were more prevalent in the brackish genomes, consistent with less possibility of using Na^+-coupled phosphate transport compared to that in the marine environment. Last, K03282, more frequently present in brackish across BM transitions, is a mechanosensitive ion channel involved in managing hypoosmotic stress (*44*).

Two distinct magnesium uptake systems (*45*, *46*) were more frequently present in lower-salinity biomes for FB and BM MSG pairs (K01531 and K03284, respectively). Moreover, for BM transitions, brackish MAGs displayed a higher presence of chemotaxis (K03413) and two-component system sensors (K02482 and K20974) involved in regulation of motility (*47*, *48*). While Mg^{2+} is a major component of sea salt (*11*) and salinity can alter chemotactic responses and motility (*49*–*51*), to the best of our knowledge, the eco-evolutionary relevance of these genes has not been previously suggested [although the differential presence of K03284 has been noted in picocyanobacteria (*52*)]. For FB, a gene connected to either pigment synthesis (K15777) or aromatic hydrocarbon degradation (K04100) was more often found in freshwater MAGs. Both of these functions may be connected to salinity-related changes in

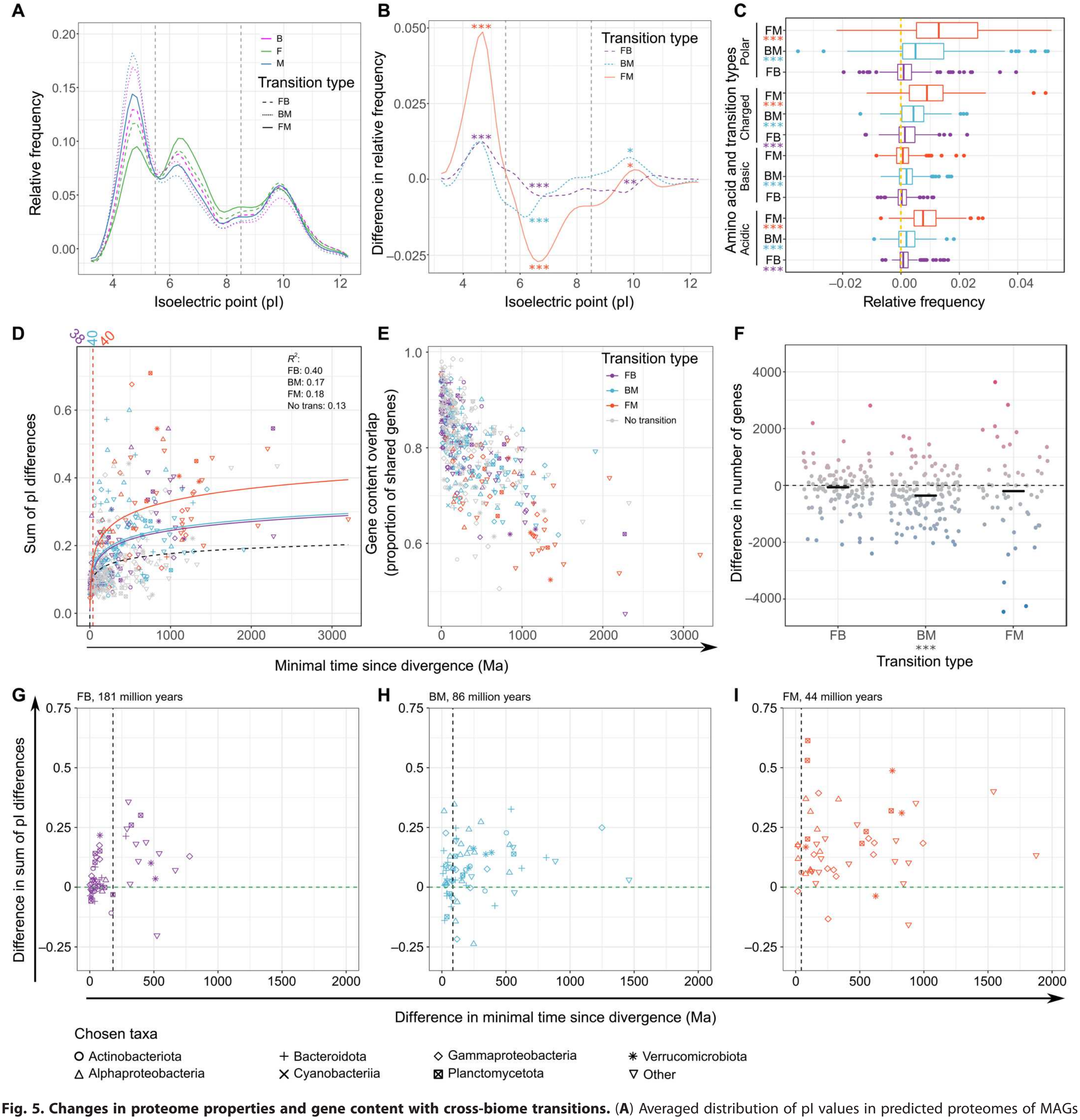

Fig. 5. Changes in proteome properties and gene content with cross-biome transitions. (**A**) Averaged distribution of pI values in predicted proteomes of MAGs calculated as mean frequencies of proteins within 0.5 pH wide bins. Vertical lines mark pI values set to distinguish between acidic, neutral, and basic proteins. (**B**) Differences in pI frequencies [as in (A)] across transitions. (**C**) Differences in fractions of the proteomes that the chosen categories of amino acids make up. The lower categories are subsets of the higher ones: polar ⊃ (charged = acidic + basic). (**D**) Sum of all differences in pI frequency versus minimal estimated divergence times, for different transition types and for no transition pairs. For no transition, the most distantly related pairs of monophyletic groups within MSGs were used. A logarithmic relationship was fitted. The vertical lines and values above correspond to the time point when the fit for a transition and no transition events diverge significantly (no overlap in ±SE ranges thereafter). (**E**) Overlap in gene content versus minimal time since divergence [grouped as in (D)]. (**F**) Differences in numbers of genes among MSG pairs. The horizontal bars represent median values. (**G** to **I**) Differences in the sum of pI differences between MSG pairs and between the biggest monophyletic groups within the MSGs formed after the transitions versus differences in minimal times since transition and subsequent within-MSG divergence events for FB (G), BM (H), and FM (I). The vertical lines correspond to the highest values in Ma ago for which the differences of sums of pI differences are not significantly higher than zero. In (B), (C), and (F), the value differences are presented as value in the more saline − value in the less saline biome; *P* values after Bonferroni correction are marked accordingly: *$P < 0.05$, **$P < 0.01$, and ***$P < 0.001$.

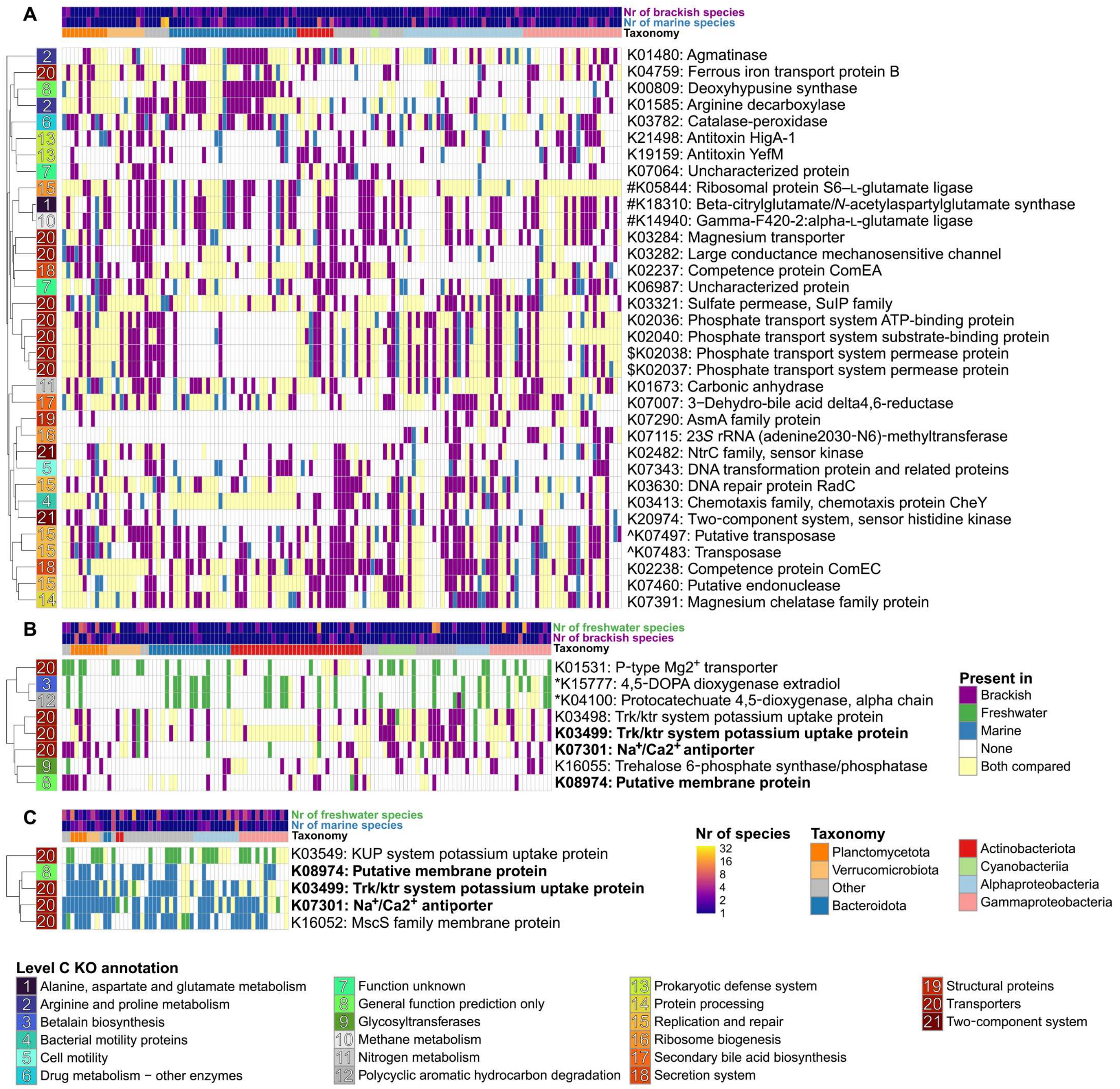

Fig. 6. Functional genes differentially present across transitions ("gain-loss maps"). Genes overrepresented in one of the biomes for MSG pairs of BM (**A**), FB (**B**), and FM (**C**). Each heatmap column is a pair of MSGs, with taxonomic annotation and the number of species in the two biomes color-coded above. Each row is an orthologous group, with its KO number and a descriptive name given to the right and a broader (KO level C) functional annotation (with color and number) to the left. To correct for labels inconsistent with the literature reference, the annotations of K01531(*45*) and K07301(*110*) were manually curated. The category "Enzymes with EC numbers" (represented by K00809) has been merged with "General function prediction only." The genes were marked as present in an MSG if they were found in >0.5 of the species within the MSG. If a gene was found in >0.5 of the representative MAGs from both MSGs across a transition, it was marked as "Both compared." Taxonomic annotation and the number of species in MSGs from respective biomes were color-coded for each transition. Only KOs found significantly (FDR-adjusted $P < 0.1$) differentially present across transitions in a pairwise analysis are displayed. Descriptions of genes identified in more than one transition type were marked in bold. Groups of genes with at least one gene being in >50% of the cases being annotated also as the other(s) were marked by adding #, $, ^, or * before the KO number. The data presented in the figure can be found together with more details in data S4.

dissolved organic carbon concentration (see Supplementary Discussion for more details).

Other differentially present genes were less directly connected to physicochemical differences between the biomes, suggesting more complex and/or cryptic biological mechanisms associated with transitions. For BM transitions, genes connected to various metabolic pathways (including modifications of charged amino acids), as well as transposases, antitoxins, and competence proteins, were more often present in brackish MAGs than their marine counterparts. Typical housekeeping genes were absent among the differentially present genes, which verifies the specificity of the method.

Last, we compared the above results with a phylogeny-unaware approach, where the presence among the bacterial species of each gene function was compared between the biomes without constraining to MSG pairs. With a few exceptions, the gene functions identified in the MSG-based analysis were also significant here (FDR-corrected $P < 0.1$) with the same patterns of differential presence, but each comparison identified thousands of additional gene functions (31 to 46% of the total number of KOs in all genomes; fig. S9 and data S4). Most of these are likely not directly linked to adaptation to the different biomes but display differential presence due to taxonomic biases. This difference provides further support to the notion that dissimilarities in gene content between bacteria inhabiting different biomes largely correspond to the selection of whole lineages (*16*) and not necessarily to functional changes allowing adaptation to a different environment.

DISCUSSION

In this study, we combined several large-scale datasets, which allowed us to comparatively analyze bacterial genomes from freshwater, brackish, and marine biomes at an unprecedented scale. Including brackish genomes allowed for a more detailed analysis of the evolutionary history of diverse aquatic bacteria than in previous studies focusing on the two biomes at the extremes of the spectrum (*9*–*11*, *16*, *43*, *53*, *54*) or specific bacterial taxa (*52*, *55*, *56*). The brackish biome is a significant part of our global waters, and combined, the two major brackish systems analyzed here have a similar volume [~99,000 km^3 (*57*)] as all freshwater lakes on earth [≤102,000 km^3 (*58*)], albeit only a fraction of the volume of the global ocean [~1.35 billion km^3 (*59*)].

We observed bacterial communities from the different biomes to be separated on at least the species level; nearly all >95% ANI genome clusters were restricted to a single biome. Brackish genome clusters often had representatives from both the Baltic Sea and Caspian Sea. These results provide further support that the brackish biome hosts a distinct, globally distributed set of bacterial species (*12*). However, the fact that populations of individual species were more genetically differentiated between the two brackish seas than across the strong salinity gradient within the Baltic Sea indicates that geographical barriers still have a structuring effect on the distribution of aquatic bacteria. The substructuring of the population from the two basins could also be affected by differences in other environmental factors, such as temperature. However, the size of the effect of geographic location in comparison to the differences across the pronounced environmental gradients of the Baltic Sea suggests that the differences can at least partially be attributed to limitations in microbial dispersal. While it might be of limited relevance on a large evolutionary time scale, it is likely to be of crucial importance when an ecosystem is disturbed, as it may take substantial amounts of time for migrating strains to fill the niches that open up as a consequence of the disturbance. Understanding how bacteria disperse and colonize new habitats is thus crucial to comprehend and mitigate the effects of such disturbances, as well as in the context of restoration efforts. For restoration, transferring of microbial communities might be a crucial component, as has been proposed for degraded soils (*60*).

Alternatively, local bacteria could adapt to environmental changes, maintaining a diverse and functioning ecosystem throughout the process. This is unlikely to be the case when it comes to changes in salinity spanning across biome barriers, however, since the formation of neither the Baltic Sea nor the Caspian Sea caused an observable increase in transition rates, which is consistent with previous notions of major geological events having little impact on bacterial speciation rates (*61*). Therefore, most of the local microdiversity that we uncovered through population genomics is likely to be lost as a consequence of such environmental changes. It has been suggested for soil microbiomes that anthropogenic pressures can lead to homogenization of communities and biodiversity loss on a global scale (*62*, *63*). Ultimately, the destruction and disturbance of environments, enhanced in the times of global change, threaten not only to decrease local microbial diversity in the short run but also to diminish genetic diversity within species at a global scale. A more detailed view of microdiversity is needed for proper protection of the genetic richness of aquatic bacteria.

Our analyses support previous concepts of FM transitions being rare (*9*–*11*) and show more common but still rare FB and BM transitions, which most often are directed toward the brackish biome. However, as the three biomes have continuously existed throughout geological history and bacterial species appear to be globally distributed within each of them (*2*), one might ask why any recent transitions have even happened. A plausible scenario may be that genetic innovation (gene gain/loss or mutation) can make a lineage competitive in a different biome, either by opening up a new niche or out-competing others in an existing one, in accordance with the stable ecotype model (*64*). However, the benefits of the innovation need to outweigh the decrease in fitness due to switching to a new salinity regime. Thus, the smaller salinity difference between the brackish and the other biomes would explain why it is associated with more transitions.

The more frequent transitions into than out of the brackish environment are harder to explain. For FB, the reason could simply be hydrological, i.e., that fresh water usually flows into brackish water rather than the other way around. For BM, differences in population sizes may instead be the explanation, as both the volume and surface of the ocean are much bigger than of all brackish waters. Beneficial mutations are thus more likely to appear among the more numerous marine bacteria. We also observed that there was a small fraction of FB and BM transitions with unusually high third-biome ancestral states (fig. S4). These events might correspond to FM transitions through brackish waters, where an intermediate period in brackish water would allow for a more gradual adaptation than between FM directly, as has been suggested before (*11*, *65*).

Our results corroborate previous reports on differences in predicted proteomes at different salinities (*14*) and support them with a systematic analysis across the bacterial tree of life. These changes likely occur over long evolutionary time scales after the actual transition, meaning that in the period after the transitions,

bacteria may have a suboptimal protein repertoire in their new environment. This environmental mismatch would potentially lower the fitness of newly transitioned lineages in addition to the bottleneck effects, traces of which are present in LD12 clade genomes (*66*) and which can lead to fixation of slightly deleterious mutations (*67*).

We observed a shift toward more acidic protein pIs at higher salinity, as also reported before (*14*, *52*, *68*). While comparison of whole metagenomes has shown a higher proportion of acidic proteins in the brackish than the marine environment (*14*), our analysis shows an opposite trend across BM transitions, consistent with previous results on picocyanobacteria (*52*) and most other close brackish and marine relatives (*14*). The discrepancy has been suggested to be caused by taxonomic biases influencing metagenome-based analysis (*14*), and we show that Actinobacteria, abundant in brackish waters (Fig. 1D), have unusually high proportions of acidic amino acids (fig. S5).

In addition, we connected the changes in isoelectric point distribution to the proteome-scale increase in proportion of acidic amino acids, most notably glutamate. This likely reflects that charged amino acids increase protein solubility at higher ionic strength (*69*, *70*). However, an even stronger pattern observed was the proportion of neutral proteins gradually diminishing with increasing salinity, which is also known from previous studies but lacks explanation (*14*). We hypothesize that this effect might be due to diminishing extracellular pH variation as the concentration of buffering salts increases (*11*, *71*). Moreover, Na^+-coupled transport is the key mechanism of pH homeostasis under alkaline conditions (*72*), to which both freshwater (*11*) and brackish environments (*71*, *73*) transcend. Consistently, lower NaCl concentrations have been connected with higher intracellular pH variation in alkaline conditions (*72*). Thus, neutral proteins could be soluble and active outside and inside the cell under pH variations in less saline environments. Unlike fresh water, brackish pH rarely changes to an acidic state (*71*, *73*), which may explain why the difference in frequency of acidic proteins was less significant for BM than for other transition types, accompanied by a change in the frequency of not only acidic but also basic amino acids.

For BM transitions, we identified genes involved in glutamate posttranslational modifications (K03412, K05844, K14940, and K18310) to be more frequent in brackish genomes. All these modifications increase the acidity of residues (*74*–*77*). Thus, depending on the specificity of the enzymes, brackish bacteria may change the charges (and pIs) of their proteins to adjust to different salinities (and varying pH). We also observed that brackish bacteria were enriched in gene functions for other physiological responses to environmental cues, including chemotaxis, transcriptional regulation, and synthesis of trehalose and polyamines (both osmolytes; see Supplementary Discussion). Thus, phenotypic plasticity might be an important hallmark of brackish bacteria, sustaining the populations across the physicochemical gradients typical for many brackish environments.

BM transitions seem to alter pangenome dynamics, as the acquisition of mobile genetic elements (MGEs) appears to be more common in brackish bacteria than their marine counterparts. This is suggested by a set of transposases [previously reported to be unusually abundant in the Baltic Sea (*78*)], competence proteins, and, indirectly, antitoxins (*79*) being more often present in the brackish MAGs (Fig. 6A). Consequently, more MGEs in the brackish MAGs could explain the higher numbers of genes as compared to the marine relatives (Fig. 5F). Moreover, MGEs tend to increase gene gain and loss processes (*80*, *81*) and may thus facilitate fast adaptation to new physiochemical conditions. This may be especially important in the brackish biome, where the environmental gradients make the niches smaller and conditions less stable. Niche generalists tend to have more open pangenomes than specialists (*82*). Gene gains and losses also promote the establishment of a recombination barrier between biomes (*83*). As homologous recombination inhibits divergence (*83*–*87*), these effects would give a further mechanistic explanation for the "species-level" separation.

In this study, we compare bacterial species for which near-complete genomes could be recovered from freshwater, brackish, and marine metagenomes. Thus, they correspond to bacterial populations notably abundant in the samples. Mapping of metagenomic reads and/or metabarcoding-based approaches could reveal that some species abundant in one biome are also present in another but in much smaller numbers. Similarly, the length of time that we estimated to have passed since all the transition events may represent them being a two-step process. It is possible that in the period following transitions, population sizes remain low in a new habitat and rise to levels allowing MAG recovery only after a considerable amount of time needed for the bacteria to further adapt to new conditions. That, however, would not change the conclusions on how the empty niches are filled after an abrupt environmental change. In addition, although the data used for this study includes two of the largest brackish water bodies, the Baltic and the Caspian seas, it does not represent the full environmental and geographic diversity of brackish waters worldwide. Expanding the analysis to include more brackish ecosystems via recently available datasets [e.g., Black Sea (*88*), Chesapeake Bay (*89*), and Pearl River Estuary (*90*)] will help in further resolving population structures and adaptations within the brackish microbiome. Moreover, while the MSG-based approach allows for classifying transitions in a meaningful way for comparative analyses without relying on ancestral state inference, it is also deemed to miss events that were followed by another cross-biome transition(s). A noteworthy example is the freshwater SAR11 (Pelagibacterales) subclade LD12, probably the best-known literature case of FM transition (*91*). The event leading to its emergence, however, is not among the transitions that we find, as its out-group is a BM MSG pair (T_218 as in data S1), corresponding to a known transition between marine and brackish biomes within SAR11 clade IIIa (*56*, *92*, *93*). Last, in the gene gain and loss analysis, we corrected for the incompleteness of the MAGs, increasing the specificity of the results. However, the subsampling procedure that we used probably also had an impact on the sensitivity of the method, and it is likely that more genes are convergently gained or lost in diverse bacteria, as they adapt to a different salinity regime. The functional annotation of the genes, as well as conclusions based on it, should be treated with caution, as the orthologs may have evolved to serve different roles in the aquatic bacteria than in the model strains.

We used a unique comparative genomics approach to systematically compare close relatives differing in categorical states. It allowed us to link evolutionary dynamics of cross biome transitions to genomic changes induced by the events through analyses that did not rely on often ambiguous ancestral state inference (which we still perform but only for inferring directions of transitions and in the process correcting for imbalances in the dataset in an evolutionary history-informed manner). This connection can be instrumental to

move beyond mere correlations of ecological and genomic changes toward their causal relationship, as we showed by unraveling the prolonged nature of posttransition proteome reorganization. While correlation-based methods can also be adjusted for a taxonomically balanced comparative genomics analysis (*94*), it is much harder to connect these results to specific evolutionary events. Furthermore, with our framework, we could correct for varying levels of genome completeness across MSG pairs and not downsampling all the MAGs to the same, lowest level, therefore minimizing the information loss. Consequently, we detected gains or losses of genes obviously related to adaptation to salinity but not any housekeeping genes. We were thus able to distill the traces of repeated gene gains and losses due to habitat change from the taxonomic effects that govern the differences in gene content of whole metagenomes (*16*). As some of the gained or lost genes have unknown functions or unobvious connections to adaptation to salinity, these results may inform investigations aiming at discoveries of new functional genes or additional functions of previously described genes.

While the method is effective in identifying genes that via horizontal transfer allows adaptation to a new environment, it will miss those that are rarely transferred but still beneficial for occupying the niches. An important example is the Na^+-transporting reduced form of nicotinamide adenine dinucleotide:ubiquinone oxidoreductase (NQR) complex. NQR functioning is directly connected to Na^+ concentrations, and its evolutionary relevance as a distinguishing feature of marine, as opposed to freshwater, bacteria has been backed by strong evidence (*11*). However, NQR is a big complex composed of many genes. Moreover, it has multiple interactions with the rest of the metabolic machinery of the cell, and how advantageous it is as an adaptation to salinity depends on the physiological setting (*11*). Thus, the conditions for the acquisition of the whole metabolic module might be rare. NQR genes were absent from the MSG-based gain/loss analysis but were among the 25 most significant ones in the phylogeny-unaware analysis for FB and FM (data S4). Together, this suggests that NQR is a feature distinguishing dominant freshwater lineages from marine and brackish ones and likely being one of the causes of the pronounced differences in taxonomic composition. However, it is not a common feature gained/lost by the newly transitioned lineages. The existence of whole metabolic modules that improve adaptation to a different environment but are unlikely to be acquired through horizontal gene transfer may be one of the barriers that cause cross-biome transitions to be rare.

Ultimately, we extend the view of transitions between aquatic biomes and highlight the brackish environment, showing trends specific for certain transition types and common across all of them. We report patterns prevalent across the bacterial tree of life, providing supplementary materials that allow investigation of the changes for chosen taxonomic groups. We observed many changes related to previously undescribed adaptation mechanisms and/or poorly characterized genes, both of which call for further exploration. In the future, phylogenomic analyses should be supplemented with experimental and ecological approaches. This would allow examining the relevance of the unraveled adaptation mechanisms in the context of ecosystem functioning and physiology.

MATERIALS AND METHODS

MAG collection and sources

Representative MAGs from freshwater, brackish, and marine biomes were collected from seven previous studies as specified in Table 1. Freshwater MAGs originate from two North American lakes (Lake Mendota and Trout Bog), 44 lakes of the StratFreshDB (mainly in Sweden, Finland, and Canada), and Lake Baikal (Russia) (*18*–*20*). Brackish MAGs originate from the Baltic Sea (*17*) and the Caspian Sea (*21*). Last, marine MAGs that were published by Tully *et al.* (*22*) and Delmont *et al.* (*23*) originate from the Tara Oceans Survey (*95*, *96*).

MAG statistics, ANI calculations, and classification

Completeness and contamination of the collected MAGs were assessed using CheckM (*97*). MAGs with ≥75% completeness and ≤5% contamination were considered for further analyses. Pairwise ANI between all MAGs was calculated using FastANI (v1.2), and MAGs were clustered at the threshold of 95% (*98*). Clustering of MAGs was done using hclust and cuttree functions in Rstudio. Taxonomic affiliation of MAGs was assigned using the "classify_wf" workflow of the GTDB Toolkit (GTDB-tk) (v 0.3.2) (*25*, *27*). To compare proportion of interbiome and brackish interbasin clusters, we used Fisher's exact test conditioned on the lower number of clusters (two categories: the basin/biome with a lower number of clusters and the number of shared clusters).

Population genomics

Population genomics was conducted with the POGENOM software as described by Sjöqvist *et al.* (*26*). The same set of 22 MAGs [from (*17*)] were used as references for mapping of metagenomic data from the Baltic (*17*) and Caspian (*21*) seas. Only five of these obtained sufficient coverage in the three Caspian Sea samples and were included in the downstream analysis. The five MAGs all represent different (>95% ANI) genome clusters. The Input_POGENOM pipeline was used for the automatic generation of input files for POGENOM. The pairwise F_{ST} values output from POGENOM were used to conduct PCoA using the Vegan package in R. Maps were plotted with the rworldmap package. For parameter settings of Input_POGENOM and POGENOM, see (*26*).

Phylogeny reconstruction

The "de_novo_wf" function of GTDB-tk (v 0.3.2) was used to reconstruct the phylogeny of MAGs (*25*, *27*). The WAG (Whelan and Goldman) amino acid evolution model was selected in the infer step, and phylum Patescibacteria was selected as the outgroup (*99*). Most downstream phylogenetic analyses were performed in RStudio (V3.6.2) with the R packages ape, phytools, phangorn and ggplot. The multi2di function in R was used to resolve multichotomies (that appear because some MAGs were nearly identical) in the phylogenetic tree. Because this function rendered some branches with zero length, we arbitrarily changed all zero-length branches to 5×10^{-4} (which was the smallest length of all other branches) to ensure that all branch lengths were >0. To remove redundant information and reduce the complexity of the phylogenetic tree, we generated a pruned tree, keeping only one MAG with the highest difference between completeness and

contamination from each cluster (or from each biome within the cluster if it contained MAGs from different biomes).

Gene prediction and annotation

Prodigal (v2.6.3) was run with default settings to identify protein-coding genes (*100*). Gene function annotation was performed with eggNOG-mapper (v2.0.0), based on the eggNOG v5.0 database (*101*, *102*). A custom Python script was used to count the number of occurrences of each KO in each annotated MAG.

Identifying MSGs for comparative analyses

Pairs of MSGs (Fig. 4A) were identified on the basis of the pruned tree. The MRCAs of the monobiomic groups were identified by choosing nodes for which on each of the two downstream branches there were MAGs from a different and only one biome. Transition types were defined by the two biomes from which the MAGs originate and do not take into consideration inferred direction of the transition. The biggest possible pairs of monophyletic groups within MSGs (coming from the same biome; only one pair for each MSG with >1 representative MAG on the pruned tree) were used as "no transition" divergence events.

Expected number of transitions

Biome annotation of the tips of the pruned tree was randomly permuted 100 times, and MSG pairs were reidentified using the same tree structure and the random biome annotations. Rounded averages of transitions obtained in all the iterations were used as the expected numbers. One-sided *P* values were calculated as the number of iterations in which a lower/higher number of transitions of a type was observed. The fractions of each transition type among all expected transitions were used as probabilities for X-squared test on the observed values.

Minimal time since divergence

Six divergence-time constraints were set as minimal estimates of time since divergence for pairs of obligate endosymbiont taxa based on fossil record of host species (table S2 and data S2) (*29*–*37*). Divergence times were calculated using RelTime (*28*) based on the unpruned trees, including GTDB reference sequences. Maximum relative rate ratio was set to 100. To correct for the difference between the placing of the root based on phylogenetic distances only (*99*) and the most probable position of the last bacterial common ancestor on the tree (*103*), Fusobacteriota were chosen as an out-group. Only the tips present in the pruned tree were chosen for further analyses.

Transition directions

One MAG from each MSG was randomly chosen. Additional MAGs from outside the MSGs were randomly chosen to downsample the dataset to the same number of genomes from each biome, equal to the lowest number for any of the biomes (396 for brackish after removing multiple MAGs within each MSG). "ace" function from the "ape" package in R was used to obtain maximum likelihood ancestral states of the MRCAs of MSG pairs using an all-rates-different model (*104*). Pairwise Wilcoxon rank-sum test of likelihoods of the ancestral states for the two biomes between which transition occurred was performed for each transition type. Random selection of MAGs within and outside the MSGs was iterated 100 times, and mean likelihoods and *P* values were used for further statistical analyses.

Comparison of isoelectric points and amino acid frequencies

Isoelectric points (pI) of all proteins within inferred bacterial proteomes were obtained using the pepstats function from the EMBOSS package (v6.6.0) (*105*). To get amino acid and amino acid category frequencies, the whole proteomes were concatenated into one sequence before running separate pepstats analyses. pI frequencies within bins of pH width of 0.5 spanning the range from 3.0 to 12.5 were used to average, plot, and compare the distributions of pIs between MSGs. Average values over MAGs (95% ANI cluster representatives) were used for MSGs consisting of more than 1 MAG.

For analysis of total changes in pI distribution, the absolute values of differences for each 0.5 pH wide bin were summed up. To compare transitions to divergence events within the same biome, within each MSG bigger than one representative MAG, a pair of monophyletic groups that diverged the longest time ago were identified. The differences between these no transition (within-MSG) monophyletic groups were compared with differences across the ancestral nodes (MRCAs of MSG pairs) in a pairwise manner. All results were tested for significance, and only the significant results are discussed in the text.

Functional gene overlap and gene numbers

Occurrences of KOs were transformed into a binary table of gene (KO) presence/absence. Gene overlap was calculated as: $\frac{N_{gS}}{(N_{g1}+N_{g2})/2}$, where N_{gS} is the number of KOs shared between 2 genomes and N_{g1} and N_{g2} are the total numbers of KOs present in the respective genomes (*83*). Formulas log(gene overlap) ~ divergence times and gene overlap ~ divergence times were used to fit logarithmic and linear models for each transition type, including no transition (see the "Comparison of isoelectric points and amino acid frequencies" section). The models were compared using a method based on the least-square means statistic (*106*). Numbers of distinct polypeptide sequences in the predicted proteomes were used as the gene numbers.

Gene presence/absence analysis

Occurrences of KOs were transformed into a binary table of gene (KO) presence/absence. Random pairs of MAGs from the MSGs were chosen for comparison for each transition type. To correct for differences in genome completeness, the presence count for the more complete MAG(s) (based on the CheckM completeness estimate) in each pair was randomly downsampled to the level of the less complete MAG. For each transition type, KOs present in at least one of the MAGs within any of the MSGs were analyzed. For each of the KOs, differences in the presence between MAGs from each combination of two biomes were analyzed using paired samples Wilcoxon test (*107*). The procedure was iterated 100 times, starting from the step of randomly choosing pairs of MAGs. The Benjamini-Hochberg method (*108*) was used to obtain FDR-adjusted *P* values (*q* values), and average *q* values from the 100 iterations for each KO were used to determine the significance of difference in each KO's presence/absence. The identified KOs were annotated using the KEGG orthology (*109*) table collected from the KEGG website on 24 November 2021.

To compare the gene content of lineages from different biomes in a phylogeny-unaware manner, we first randomly downsampled the genes present/absent in all the representative MAGs of identified bacterial species (i.e., the MAGs of the pruned tree) to 75% completeness level. Then, we assessed the presence/absence of each gene in a pair of biomes using unpaired Wilcoxon test.

Statistical analyses

If not stated otherwise, pairwise Wilcoxon rank-sum test (*107*) was used to compare values across MSG pairs of a certain type. If not stated otherwise, family-wise error rate ≤0.05 was considered as the threshold of significance. The analyses were performed using R.

Supplementary Materials

This PDF file includes:
Tables S1 to S3
Figs. S1 to S9
Supplementary Discussion
Legends for data S1 to S4
References

Other Supplementary Material for this manuscript includes the following:
Data S1 to S4

View/request a protocol for this paper from *Bio-protocol*.

REFERENCES AND NOTES

1. N. Eldredge, S. J. Gould, Punctuated equilibria: An alternative to phyletic gradualism, in *Models in Paleobiology* (Freeman Cooper & Co, 1972); http://georgealozano.com/teach/evolution-UNBC/papers/eldredge.pdf.
2. S. Louca, The rates of global bacterial and archaeal dispersal. *ISME J.* **16**, 159–167 (2022).
3. P. Puigbò, A. E. Lobkovsky, D. M. Kristensen, Y. I. Wolf, E. V. Koonin, Genomes in turmoil: Quantification of genome dynamics in prokaryote supergenomes. *BMC Biol.* **12**, 66 (2014).
4. M. Vos, M. C. Hesselman, T. A. Te Beek, M. W. J. van Passel, A. Eyre-Walker, Rates of lateral gene transfer in prokaryotes: High but why? *Trends Microbiol.* **23**, 598–605 (2015).
5. Intergovernmental Panel on Climate Change, Coastal systems and low-lying areas, in *Climate Change 2014—Impacts, Adaptation and Vulnerability: Part A: Global and Sectoral Aspects: Working Group II Contribution to the IPCC Fifth Assessment Report* (Cambridge Univ. Press, 2014), pp. 361–410.
6. P. G. Falkowski, T. Fenchel, E. F. Delong, The microbial engines that drive Earth's biogeochemical cycles. *Science* **320**, 1034–1039 (2008).
7. S. S. Ali, Y. Yu, M. Pfosser, W. Wetschnig, Inferences of biogeographical histories within subfamily Hyacinthoideae using S-DIVA and Bayesian binary MCMC analysis implemented in RASP (Reconstruct Ancestral State in Phylogenies). *Ann. Bot.* **109**, 95–107 (2012).
8. L. R. Thompson, J. G. Sanders, D. McDonald, A. Amir, J. Ladau, K. J. Locey, R. J. Prill, A. Tripathi, S. M. Gibbons, G. Ackermann, J. A. Navas-Molina, S. Janssen, E. Kopylova, Y. Vázquez-Baeza, A. González, J. T. Morton, S. Mirarab, Z. Zech Xu, L. Jiang, M. F. Haroon, J. Kanbar, Q. Zhu, S. Jin Song, T. Kosciolek, N. A. Bokulich, J. Lefler, C. J. Brislawn, G. Humphrey, S. M. Owens, J. Hampton-Marcell, D. Berg-Lyons, V. McKenzie, N. Fierer, J. A. Fuhrman, A. Clauset, R. L. Stevens, A. Shade, K. S. Pollard, K. D. Goodwin, J. K. Jansson, J. A. Gilbert, R. Knight; Earth Microbiome Project Consortium, A communal catalogue reveals Earth's multiscale microbial diversity. *Nature* **551**, 457–463 (2017).
9. R. Logares, J. Bråte, S. Bertilsson, J. L. Clasen, K. Shalchian-Tabrizi, K. Rengefors, Infrequent marine-freshwater transitions in the microbial world. *Trends Microbiol.* **17**, 414–422 (2009).
10. A. Barberán, E. O. Casamayor, Global phylogenetic community structure and β-diversity patterns in surface bacterioplankton metacommunities. *Aquat. Microb. Ecol.* **59**, 1–10 (2010).
11. D. A. Walsh, J. Lafontaine, H.-P. Grossart, On the eco-evolutionary relationships of fresh and salt water bacteria and the role of gene transfer in their adaptation, in *Lateral Gene Transfer in Evolution*, U. Gophna, Ed. (Springer, 2013), pp. 55–77.
12. L. W. Hugerth, J. Larsson, J. Alneberg, M. V. Lindh, C. Legrand, J. Pinhassi, A. F. Andersson, Metagenome-assembled genomes uncover a global brackish microbiome. *Genome Biol.* **16**, 279 (2015).
13. L. F. Delgado, A. F. Andersson, Evaluating metagenomic assembly approaches for biome-specific gene catalogues. *Microbiome* **10**, 72 (2022).
14. P. J. Cabello-Yeves, F. Rodriguez-Valera, Marine-freshwater prokaryotic transitions require extensive changes in the predicted proteome. *Microbiome* **7**, 117 (2019).
15. C. L. Dupont, J. Larsson, S. Yooseph, K. Ininbergs, J. Goll, J. Asplund-Samuelsson, J. P. McCrow, N. Celepli, L. Z. Allen, M. Ekman, A. J. Lucas, Å. Hagström, M. Thiagarajan, B. Brindefalk, A. R. Richter, A. F. Andersson, A. Tenney, D. Lundin, A. Tovchigrechko, J. A. A. Nylander, D. Brami, J. H. Badger, A. E. Allen, D. B. Rusch, J. Hoffman, E. Norrby, R. Friedman, J. Pinhassi, J. C. Venter, B. Bergman, Functional tradeoffs underpin salinity-driven divergence in microbial community composition. *PLOS ONE* **9**, e89549 (2014).
16. A. Eiler, K. Zaremba-Niedzwiedzka, M. Martínez-García, K. D. McMahon, R. Stepanauskas, S. G. E. Andersson, S. Bertilsson, Productivity and salinity structuring of the microplankton revealed by comparative freshwater metagenomics. *Environ. Microbiol.* **16**, 2682–2698 (2014).
17. J. Alneberg, C. Bennke, S. Beier, C. Bunse, C. Quince, K. Ininbergs, L. Riemann, M. Ekman, K. Jürgens, M. Labrenz, J. Pinhassi, A. F. Andersson, Ecosystem-wide metagenomic binning enables prediction of ecological niches from genomes. *Commun Biol.* **3**, 119 (2020).
18. A. M. Linz, S. He, S. L. R. Stevens, K. Anantharaman, R. R. Rohwer, R. R. Malmstrom, S. Bertilsson, K. D. McMahon, Freshwater carbon and nutrient cycles revealed through reconstructed population genomes. *PeerJ.* **6**, e6075 (2018).
19. P. J. Cabello-Yeves, T. I. Zemskaya, R. Rosselli, F. H. Coutinho, A. S. Zakharenko, V. V. Blinov, F. Rodriguez-Valera, Genomes of novel microbial lineages assembled from the sub-ice waters of Lake Baikal. *Appl. Environ. Microbiol.* **84**, e02132-17 (2017).
20. M. Buck, S. L. Garcia, L. Fernandez, G. Martin, G. A. Martinez-Rodriguez, J. Saarenheimo, J. Zopfi, S. Bertilsson, S. Peura, Comprehensive dataset of shotgun metagenomes from oxygen stratified freshwater lakes and ponds. *Sci Data.* **8**, 131 (2021).
21. M. Mehrshad, M. A. Amoozegar, R. Ghai, S. A. Shahzadeh Fazeli, F. Rodriguez-Valera, Genome reconstruction from metagenomic data sets reveals novel microbes in the brackish waters of the Caspian Sea. *Appl. Environ. Microbiol.* **82**, 1599–1612 (2016).
22. B. J. Tully, E. D. Graham, J. F. Heidelberg, The reconstruction of 2,631 draft metagenome-assembled genomes from the global oceans. *Sci Data.* **5**, 170203 (2018).
23. T. O. Delmont, C. Quince, A. Shaiber, Ö. C. Esen, S. T. Lee, M. S. Rappé, S. L. McLellan, S. Lücker, A. M. Eren, Nitrogen-fixing populations of Planctomycetes and Proteobacteria are abundant in surface ocean metagenomes. *Nat. Microbiol.* **3**, 804–813 (2018).
24. N. J. Varghese, S. Mukherjee, N. Ivanova, K. T. Konstantinidis, K. Mavrommatis, N. C. Kyrpides, A. Pati, Microbial species delineation using whole genome sequences. *Nucleic Acids Res.* **43**, 6761–6771 (2015).
25. D. H. Parks, M. Chuvochina, P.-A. Chaumeil, C. Rinke, A. J. Mussig, P. Hugenholtz, A complete domain-to-species taxonomy for Bacteria and Archaea. *Nat. Biotechnol.* **38**, 1079–1086 (2020).
26. C. Sjöqvist, L. F. Delgado, J. Alneberg, A. F. Andersson, Ecologically coherent population structure of uncultivated bacterioplankton. *ISME J.* **15**, 3034–3049 (2021).
27. P.-A. Chaumeil, A. J. Mussig, P. Hugenholtz, D. H. Parks, GTDB-Tk: A toolkit to classify genomes with the Genome Taxonomy Database. *Bioinformatics* **36**, 1925–1927 (2019).
28. F. U. Battistuzzi, Q. Tao, L. Jones, K. Tamura, S. Kumar, RelTime relaxes the strict molecular clock throughout the phylogeny. *Genome Biol. Evol.* **10**, 1631–1636 (2018).
29. H. Kim, S. Lee, Y. Jang, Macroevolutionary patterns in the Aphidini aphids (Hemiptera: Aphididae): Diversification, host association, and biogeographic origins. *PLOS ONE* **6**, e24749 (2011).
30. N. A. Moran, M. A. Munson, P. Baumann, H. Ishikawa, A molecular clock in endosymbiotic bacteria is calibrated using the insect hosts. *Proc. R. Soc. Lond. B Biol. Sci.* **253**, 167–171 (1993).
31. M. L. Thao, N. A. Moran, P. Abbot, E. B. Brennan, D. H. Burckhardt, P. Baumann, Cospeciation of psyllids and their primary prokaryotic endosymbionts. *Appl. Environ. Microbiol.* **66**, 2898–2905 (2000).
32. T. D. A. Cockerell, The third fossil tsetse-fly. *Nature* **98**, 70 (1916).
33. N. Lo, C. Bandi, H. Watanabe, C. Nalepa, T. Beninati, Evidence for cocladogenesis between diverse dictyopteran lineages and their intracellular endosymbionts. *Mol. Biol. Evol.* **20**, 907–913 (2003).
34. B. L. Thorne, D. A. Grimaldi, K. Krishna, Early fossil history of the termites, in *Termites: Evolution, Sociality, Symbioses, Ecology*, T. Abe, D. E. Bignell, M. Higashi, Eds. (Springer, 2000), pp. 77–93.
35. N. A. Moran, P. Tran, N. M. Gerardo, Symbiosis and insect diversification: An ancient symbiont of sap-feeding insects from the bacterial phylum Bacteroidetes. *Appl. Environ. Microbiol.* **71**, 8802–8810 (2005).

36. D. Shcherbakov, The 270 million year history of Auchenorrhyncha (Homoptera). *Denisia* **176**, 29–36 (2002).
37. C.-H. Kuo, H. Ochman, Inferring clocks when lacking rocks: The variable rates of molecular evolution in bacteria. *Biol. Direct* **4**, 35 (2009).
38. S. Björck, A review of the history of the Baltic Sea, 13.0-8.0 ka BP. *Quat. Int.* **27**, 19–40 (1995).
39. H. J. Dumont, The Caspian Lake: History, biota, structure, and function. *Limnol. Oceanogr.* **43**, 44–52 (1998).
40. K. L. Morrison, G. A. Weiss, Combinatorial alanine-scanning. *Curr. Opin. Chem. Biol.* **5**, 302–307 (2001).
41. D. Bossemeyer, A. Borchard, D. C. Dosch, G. C. Helmer, W. Epstein, I. R. Booth, E. P. Bakker, K+-transport protein TrkA of Escherichia coli is a peripheral membrane protein that requires other trk Gene products for attachment to the cytoplasmic membrane. *J. Biol. Chem.* **264**, 16403–16410 (1989).
42. G. Holtmann, E. P. Bakker, N. Uozumi, E. Bremer, KtrAB and KtrCD: Two K+ uptake systems in Bacillus subtilis and their role in adaptation to hypertonicity. *J. Bacteriol.* **185**, 1289–1298 (2003).
43. H. Zhang, S. Yoshizawa, Y. Sun, Y. Huang, X. Chu, J. M. González, J. Pinhassi, H. Luo, Repeated evolutionary transitions of flavobacteria from marine to non-marine habitats. *Environ. Microbiol.* **21**, 648–666 (2019).
44. N. Levina, S. Tötemeyer, N. R. Stokes, P. Louis, M. A. Jones, I. R. Booth, Protection of Escherichia coli cells against extreme turgor by activation of MscS and MscL mechanosensitive channels: Identification of genes required for MscS activity. *EMBO J.* **18**, 1730–1737 (1999).
45. T. Tao, M. D. Snavely, S. G. Farr, M. E. Maguire, Magnesium transport in Salmonella typhimurium: mgtA encodes a P-type ATPase and is regulated by Mg^{2+} in a manner similar to that of the mgtB P-type ATPase. *J. Bacteriol.* **177**, 2654–2662 (1995).
46. K. M. Papp, M. E. Maguire, The CorA Mg^{2+} transporter does not transport Fe^{2+}. *J. Bacteriol.* **186**, 7653–7658 (2004).
47. J.-L. Hsu, H.-C. Chen, H.-L. Peng, H.-Y. Chang, Characterization of the histidine-containing phosphotransfer protein B-mediated multistep phosphorelay system in Pseudomonas aeruginosa PAO1. *J. Biol. Chem.* **283**, 9933–9944 (2008).
48. M. M. S. M. Wösten, J. A. Wagenaar, J. P. M. van Putten, The FlgS/FlgR two-component signal transduction system regulates the fla regulon in Campylobacter jejuni. *J. Biol. Chem.* **279**, 16214–16222 (2004).
49. H. Futamata, M. Sakai, H. Ozawa, Y. Urashima, T. Sueguchi, T. Matsuguchi, Chemotactic response to amino acids of fluorescent pseudomonads isolated from spinach roots grown in soils with different salinity levels. *Soil Sci. Plant Nutr.* **44**, 1–7 (1998).
50. M. H. Larsen, N. Blackburn, J. L. Larsen, J. E. Olsen, Influences of temperature, salinity and starvation on the motility and chemotactic response of Vibrio anguillarum. *Microbiology* **150**, 1283–1290 (2004).
51. X. Xu, H. Li, X. Qi, Y. Chen, Y. Qin, J. Zheng, X. Jiang, *cheA*, *cheB*, *cheR*, *cheV*, and *cheY* are involved in regulating the adhesion of *Vibrio harveyi*. *Front. Cell. Infect. Microbiol.* **10**, 591751 (2020).
52. P. J. Cabello-Yeves, C. Callieri, A. Picazo, L. Schallenberg, P. Huber, J. J. Roda-Garcia, M. Bartosiewicz, O. I. Belykh, I. V. Tikhonova, A. Torcello-Requena, P. M. De Prado, R. J. Puxty, A. D. Millard, A. Camacho, F. Rodriguez-Valera, D. J. Scanlan, Elucidating the picocyanobacteria salinity divide through ecogenomics of new freshwater isolates. *BMC Biol.* **20**, 175 (2022).
53. S. F. Paver, D. Muratore, R. J. Newton, M. L. Coleman, Reevaluating the salty divide: Phylogenetic specificity of transitions between marine and freshwater systems. *mSystems* **3**, e00232-18 (2018).
54. A. Ramachandran, S. McLatchie, D. A. Walsh, A novel freshwater to marine evolutionary transition revealed within *Methylophilaceae* bacteria from the Arctic Ocean. *MBio* **12**, e0130621 (2021).
55. A. Eiler, R. Mondav, L. Sinclair, L. Fernandez-Vidal, D. G. Scofield, P. Schwientek, M. Martinez-Garcia, D. Torrents, K. D. McMahon, S. G. Andersson, R. Stepanauskas, T. Woyke, S. Bertilsson, Tuning fresh: Radiation through rewiring of central metabolism in streamlined bacteria. *ISME J.* **10**, 1902–1914 (2016).
56. V. C. Lanclos, A. N. Rasmussen, C. Y. Kojima, C. Cheng, M. W. Henson, B. C. Faircloth, C. A. Francis, J. C. Thrash, Ecophysiology and genomics of the brackish water adapted SAR11 subclade IIIa. *ISME J.* **17**, 620–629 (2023).
57. P. Snoeijs-Leijonmalm, E. Andrén, Why is the Baltic Sea so special to live in?, in *Biological Oceanography of the Baltic Sea*, P. Snoeijs-Leijonmalm, H. Schubert, T. Radziejewska, Eds. (Springer, 2017), pp. 23–84.
58. P. H. Gleick, *Water in Crisis: A Guide to the World's Fresh Water Resources* (Oxford Univ. Press, 1993).
59. T. S. Garrison, *Oceanography: An Invitation to Marine Science* (Cengage Learning, 2012).
60. O. Coban, G. B. De Deyn, M. van der Ploeg, Soil microbiota as game-changers in restoration of degraded lands. *Science* **375**, abe0725 (2022).
61. S. Louca, P. M. Shih, M. W. Pennell, W. W. Fischer, L. W. Parfrey, M. Doebeli, Bacterial diversification through geological time. *Nat Ecol Evol.* **2**, 1458–1467 (2018).
62. C. A. Guerra, M. Delgado-Baquerizo, E. Duarte, O. Marigliano, C. Görgen, F. T. Maestre, N. Eisenhauer, Global projections of the soil microbiome in the Anthropocene. *Glob. Ecol. Biogeogr.* **30**, 987–999 (2021).
63. C. Averill, M. A. Anthony, P. Baldrian, F. Finkbeiner, J. van den Hoogen, T. Kiers, P. Kohout, E. Hirt, G. R. Smith, T. W. Crowther, Defending Earth's terrestrial microbiome. *Nat. Microbiol.* **7**, 1717–1725 (2022).
64. F. M. Cohan, Bacterial species and speciation. *Syst. Biol.* **50**, 513–524 (2001).
65. J. Kan, S. E. Evans, F. Chen, M. T. Suzuki, Novel estuarine bacterioplankton in rRNA operon libraries from the Chesapeake Bay. *Aquat. Microb. Ecol.* **51**, 55–66 (2008).
66. K. Zaremba-Niedzwiedzka, J. Viklund, W. Zhao, J. Ast, A. Sczyrba, T. Woyke, K. McMahon, S. Bertilsson, R. Stepanauskas, S. G. E. Andersson, Single-cell genomics reveal low recombination frequencies in freshwater bacteria of the SAR11 clade. *Genome Biol.* **14**, R130 (2013).
67. T. Ohta, Slightly deleterious mutant substitutions in evolution. *Nature* **246**, 96–98 (1973).
68. J. Kiraga, P. Mackiewicz, D. Mackiewicz, M. Kowalczuk, P. Biecek, N. Polak, K. Smolarczyk, M. R. Dudek, S. Cebrat, The relationships between the isoelectric point and: length of proteins, taxonomy and ecology of organisms. *BMC Genomics* **8**, 163 (2007).
69. J. K. Lanyi, Salt-dependent properties of proteins from extremely halophilic bacteria. *Bacteriol. Rev.* **38**, 272–290 (1974).
70. M. Mevarech, F. Frolow, L. M. Gloss, Halophilic enzymes: Proteins with a grain of salt. *Biophys. Chem.* **86**, 155–164 (2000).
71. J. Carstensen, C. M. Duarte, Drivers of pH variability in coastal ecosystems. *Environ. Sci. Technol.* **53**, 4020–4029 (2019).
72. J. L. Slonczewski, M. Fujisawa, M. Dopson, T. A. Krulwich, Cytoplasmic pH measurement and homeostasis in Bacteria and Archaea, in *Advances in Microbial Physiology*, R. K. Poole, Ed. (Academic Press, 2009), vol. 55, pp. 1–317.
73. B. Schneider, O. Dellwig, K. Kuliński, A. Omstedt, F. Pollehne, G. Rehder, O. Savchuk, Biogeochemical cycles, in *Biological Oceanography of the Baltic Sea*, P. Snoeijs-Leijonmalm, H. Schubert, T. Radziejewska, Eds. (Springer, 2017), pp. 87–122.
74. M. L. Kirsch, P. D. Peters, D. W. Hanlon, J. R. Kirby, G. W. Ordal, Chemotactic methylesterase promotes adaptation to high concentrations of attractant in Bacillus subtilis. *J. Biol. Chem.* **268**, 18610–18616 (1993).
75. F. Collard, V. Stroobant, P. Lamosa, C. N. Kapanda, D. M. Lambert, G. G. Muccioli, J. H. Poupaert, F. Opperdoes, E. Van Schaftingen, Molecular identification of N-acetylaspartylglutamate synthase and β-citrylglutamate synthase. *J. Biol. Chem.* **285**, 29826–29833 (2010).
76. H. Li, H. Xu, D. E. Graham, R. H. White, Glutathione synthetase homologs encode α-l-glutamate ligases for methanogenic coenzyme F_{420} and tetrahydrosarcinapterin biosyntheses. *Proc. Natl. Acad. Sci. U.S.A.* **100**, 9785–9790 (2003).
77. K. Kino, T. Arai, Y. Arimura, Poly-α-glutamic acid synthesis using a novel catalytic activity of RimK from Escherichia coli K-12. *Appl. Environ. Microbiol.* **77**, 2019–2025 (2011).
78. T. Vigil-Stenman, K. Ininbergs, B. Bergman, M. Ekman, High abundance and expression of transposases in bacteria from the Baltic Sea. *ISME J.* **11**, 2611–2623 (2017).
79. D. J. Rankin, E. P. C. Rocha, S. P. Brown, What traits are carried on mobile genetic elements, and why? *Heredity* **106**, 1–10 (2011).
80. E. V. Koonin, Y. I. Wolf, Genomics of bacteria and archaea: The emerging dynamic view of the prokaryotic world. *Nucleic Acids Res.* **36**, 6688–6719 (2008).
81. S. M. Soucy, J. Huang, J. P. Gogarten, Horizontal gene transfer: Building the web of life. *Nat. Rev. Genet.* **16**, 472–482 (2015).
82. M. A. Brockhurst, E. Harrison, J. P. J. Hall, T. Richards, A. McNally, C. MacLean, The ecology and evolution of pangenomes. *Curr. Biol.* **29**, R1094–R1103 (2019).
83. J. Iranzo, Y. I. Wolf, E. V. Koonin, I. Sela, Gene gain and loss push prokaryotes beyond the homologous recombination barrier and accelerate genome sequence divergence. *Nat. Commun.* **10**, 5376 (2019).
84. M. Hoetzinger, M. W. Hahn, Genomic divergence and cohesion in a species of pelagic freshwater bacteria. *BMC Genomics* **18**, 794 (2017).
85. W. P. Hanage, B. G. Spratt, K. M. E. Turner, C. Fraser, Modelling bacterial speciation. *Philos. Trans. R. Soc. Lond. B Biol. Sci.* **361**, 2039–2044 (2006).
86. P. Marttinen, N. J. Croucher, M. U. Gutmann, J. Corander, W. P. Hanage, Recombination produces coherent bacterial species clusters in both core and accessory genomes. *Microb. Genom.* **1**, e000038 (2015).
87. R. J. Whitaker, D. W. Grogan, J. W. Taylor, Recombination shapes the natural population structure of the hyperthermophilic archaeon *Sulfolobus islandicus*. *Mol. Biol. Evol.* **22**, 2354–2361 (2005).

88. P. J. Cabello-Yeves, C. Callieri, A. Picazo, M. Mehrshad, J. M. Haro-Moreno, J. J. Roda-Garcia, N. Dzhembekova, V. Slabakova, N. Slabakova, S. Moncheva, F. Rodriguez-Valera, The microbiome of the Black Sea water column analyzed by shotgun and genome centric metagenomics. *Environ. Microbiome.* **16**, 5 (2021).
89. M. A. Ahmed, S. J. Lim, B. J. Campbell, Metagenomes, metatranscriptomes, and metagenome-assembled genomes from Chesapeake and Delaware Bay (USA) water samples. *Microbiol. Resour. Announc.* **10**, e0026221 (2021).
90. B. Xu, F. Li, L. Cai, R. Zhang, L. Fan, C. Zhang, A holistic genome dataset of bacteria, archaea and viruses of the Pearl River estuary. *Sci. Data.* **9**, 49 (2022).
91. R. Logares, J. Bråte, F. Heinrich, K. Shalchian-Tabrizi, S. Bertilsson, Infrequent transitions between saline and fresh waters in one of the most abundant microbial lineages (SAR11). *Mol. Biol. Evol.* **27**, 347–357 (2010).
92. D. P. R. Herlemann, J. Woelk, M. Labrenz, K. Jürgens, Diversity and abundance of "Pelagibacterales" (SAR11) in the Baltic Sea salinity gradient. *Syst. Appl. Microbiol.* **37**, 601–604 (2014).
93. M. W. Henson, V. C. Lanclos, D. M. Pitre, J. L. Weckhorst, A. M. Lucchesi, C. Cheng, B. Temperton, J. C. Thrash, Expanding the diversity of Bacterioplankton isolates and modeling isolation efficacy with large-scale dilution-to-extinction cultivation. *Appl. Environ. Microbiol.* **86**, e00943-20 (2020).
94. A. R. Ives, T. Garland Jr., Phylogenetic logistic regression for binary dependent variables. *Syst. Biol.* **59**, 9–26 (2010).
95. S. Sunagawa, S. G. Acinas, P. Bork, C. Bowler; Tara Oceans Coordinators, D. Eveillard, G. Gorsky, L. Guidi, D. Iudicone, E. Karsenti, F. Lombard, H. Ogata, S. Pesant, M. B. Sullivan, P. Wincker, C. de Vargas, Tara Oceans: Towards global ocean ecosystems biology. *Nat. Rev. Microbiol.* **18**, 428–445 (2020).
96. D. B. Rusch, A. L. Halpern, G. Sutton, K. B. Heidelberg, S. Williamson, S. Yooseph, D. Wu, J. A. Eisen, J. M. Hoffman, K. Remington, K. Beeson, B. Tran, H. Smith, H. Baden-Tillson, C. Stewart, J. Thorpe, J. Freeman, C. Andrews-Pfannkoch, J. E. Venter, K. Li, S. Kravitz, J. F. Heidelberg, T. Utterback, Y.-H. Rogers, L. I. Falcón, V. Souza, G. Bonilla-Rosso, L. E. Eguiarte, D. M. Karl, S. Sathyendranath, T. Platt, E. Bermingham, V. Gallardo, G. Tamayo-Castillo, M. R. Ferrari, R. L. Strausberg, K. Nealson, R. Friedman, M. Frazier, J. C. Venter, The Sorcerer II Global Ocean Sampling expedition: Northwest Atlantic through eastern tropical Pacific. *PLOS Biol.* **5**, e77 (2007).
97. D. H. Parks, M. Imelfort, C. T. Skennerton, P. Hugenholtz, G. W. Tyson, CheckM: Assessing the quality of microbial genomes recovered from isolates, single cells, and metagenomes. *Genome Res.* **25**, 1043–1055 (2015).
98. C. Jain, L. M. Rodriguez-R, A. M. Phillippy, K. T. Konstantinidis, S. Aluru, High throughput ANI analysis of 90K prokaryotic genomes reveals clear species boundaries. *Nat. Commun.* **9**, 5114 (2018).
99. Q. Zhu, U. Mai, W. Pfeiffer, S. Janssen, F. Asnicar, J. G. Sanders, P. Belda-Ferre, G. A. Al-Ghalith, E. Kopylova, D. McDonald, T. Kosciolek, J. B. Yin, S. Huang, N. Salam, J.-Y. Jiao, Z. Wu, Z. Z. Xu, K. Cantrell, Y. Yang, E. Sayyari, M. Rabiee, J. T. Morton, S. Podell, D. Knights, W.-J. Li, C. Huttenhower, N. Segata, L. Smarr, S. Mirarab, R. Knight, Phylogenomics of 10,575 genomes reveals evolutionary proximity between domains Bacteria and Archaea. *Nat. Commun.* **10**, 5477 (2019).
100. D. Hyatt, G.-L. Chen, P. F. Locascio, M. L. Land, F. W. Larimer, L. J. Hauser, Prodigal: Prokaryotic gene recognition and translation initiation site identification. *BMC Bioinformatics.* **11**, 119 (2010).
101. J. Huerta-Cepas, K. Forslund, L. P. Coelho, D. Szklarczyk, L. J. Jensen, C. von Mering, P. Bork, Fast genome-wide functional annotation through orthology assignment by eggNOG-Mapper. *Mol. Biol. Evol.* **34**, 2115–2122 (2017).
102. J. Huerta-Cepas, D. Szklarczyk, D. Heller, A. Hernández-Plaza, S. K. Forslund, H. Cook, D. R. Mende, I. Letunic, T. Rattei, L. J. Jensen, C. von Mering, P. Bork, eggNOG 5.0: A hierarchical, functionally and phylogenetically annotated orthology resource based on 5090 organisms and 2502 viruses. *Nucleic Acids Res.* **47**, D309–D314 (2019).
103. G. A. Coleman, A. A. Davín, T. A. Mahendrarajah, L. L. Szánthó, A. Spang, P. Hugenholtz, G. J. Szöllősi, T. A. Williams, A rooted phylogeny resolves early bacterial evolution. *Science* 372, eabe0511 (2021), doi:10.1126/science.abe0511.
104. M. Pagel, Detecting correlated evolution on phylogenies: A general method for the comparative analysis of discrete characters. *Proc. R. Soc. Lond. B Biol. Sci.* **255**, 37–45 (1994).
105. P. Rice, I. Longden, A. Bleasby, EMBOSS: The European Molecular Biology Open Software Suite. *Trends Genet.* **16**, 276–277 (2000).
106. R. V. Lenth, Least-squares means: The R Package lsmeans. *J. Stat. Softw.* **69**, 1–33 (2016).
107. F. Wilcoxon, Individual comparisons by ranking methods. *Biometrics* **1**, 80–83 (1945).
108. Y. Benjamini, Y. Hochberg, Controlling the false discovery rate: A practical and powerful approach to multiple testing. *J. R. Stat. Soc.* **57**, 289–300 (1995).
109. M. Kanehisa, Y. Sato, M. Kawashima, M. Furumichi, M. Tanabe, KEGG as a reference resource for gene and protein annotation. *Nucleic Acids Res.* **44**, D457–D462 (2016).
110. G. M. Besserer, D. A. Nicoll, J. Abramson, K. D. Philipson, Characterization and purification of a Na^+/Ca^{2+} exchanger from an Archaebacterium. *J. Biol. Chem.* **287**, 8652–8659 (2012).
111. M. E. Maguire, Magnesium transporters: Properties, regulation and structure. *Front. Biosci.* **11**, 3149–3163 (2006).
112. F. Gandía-Herrero, F. García-Carmona, Escherichia coli protein YgiD produces the structural unit of plant pigments betalains: Characterization of a prokaryotic enzyme with DOPA-extradiol-dioxygenase activity. *Appl. Microbiol. Biotechnol.* **98**, 1165–1174 (2014).
113. F. Gandía-Herrero, J. Escribano, F. García-Carmona, Structural implications on color, fluorescence, and antiradical activity in betalains. *Planta* **232**, 449–460 (2010).
114. T. W. Ghylin, S. L. Garcia, F. Moya, B. O. Oyserman, P. Schwientek, K. T. Forest, J. Mutschler, J. Dwulit-Smith, L.-K. Chan, M. Martinez-Garcia, A. Sczyrba, R. Stepanauskas, H.-P. Grossart, T. Woyke, F. Warnecke, R. Malmstrom, S. Bertilsson, K. D. McMahon, Comparative single-cell genomics reveals potential ecological niches for the freshwater acI Actinobacteria lineage. *ISME J.* **8**, 2503–2516 (2014).
115. J. D. Lipscomb, Mechanism of extradiol aromatic ring-cleaving dioxygenases. *Curr. Opin. Struct. Biol.* **18**, 644–649 (2008).
116. L. A. Mueller, U. Hinz, J.-P. Zryd, The formation of betalamic acid and muscaflavin by recombinant dopa-dioxygenase from *Amanita*. *Phytochemistry* **44**, 567–569 (1997).
117. M. A. Guerrero-Rubio, F. García-Carmona, F. Gandía-Herrero, First description of betalains biosynthesis in an aquatic organism: Characterization of 4,5-DOPA-extradiol-dioxygenase activity in the cyanobacteria Anabaena cylindrica. *J. Microbial. Biotechnol.* **13**, 1948–1959 (2020).
118. J. Hill, E. D. Enbody, M. E. Pettersson, C. G. Sprehn, D. Bekkevold, A. Folkvord, L. Laikre, G. Kleinau, P. Scheerer, L. Andersson, Recurrent convergent evolution at amino acid residue 261 in fish rhodopsin. *Proc. Natl. Acad. Sci. U.S.A.* **116**, 18473–18478 (2019).
119. Y. Noda, S. Nishikawa, K. Shiozuka, H. Kadokura, H. Nakajima, K. Yoda, Y. Katayama, N. Morohoshi, T. Haraguchi, M. Yamasaki, Molecular cloning of the protocatechuate 4,5-dioxygenase genes of Pseudomonas paucimobilis. *J. Bacteriol.* **172**, 2704–2709 (1990).
120. N. Kamimura, E. Masai, The Protocatechuate 4,5-cleavage pathway: overview and new findings, in *Biodegradative Bacteria: How Bacteria Degrade, Survive, Adapt, and Evolve*, H. Nojiri, M. Tsuda, M. Fukuda, Y. Kamagata, Eds. (Springer, 2014), pp. 207–226.
121. E. Tsagogiannis, E. Vandera, A. Primikyri, S. Asimakoula, A. G. Tzakos, I. P. Gerothanassis, A.-I. Koukkou, Characterization of Protocatechuate 4,5-dioxygenase from *Pseudarthrobacter phenanthrenivorans* Sphe3 and in situ reaction monitoring in the NMR tube. *Int. J. Mol. Sci.* **22**, 9647 (2021).
122. A. M. Burroughs, M. E. Glasner, K. P. Barry, E. A. Taylor, L. Aravind, Oxidative opening of the aromatic ring: Tracing the natural history of a large superfamily of dioxygenase domains and their relatives. *J. Biol. Chem.* **294**, 10211–10235 (2019).
123. S. Berry, B. Esper, I. Karandashova, M. Teuber, I. Elanskaya, M. Rögner, M. Hagemann, Potassium uptake in the unicellular cyanobacterium Synechocystis sp. strain PCC 6803 mainly depends on a Ktr-like system encoded by slr1509 (ntpJ). *FEBS Lett.* **548**, 53–58 (2003).
124. K. Nanatani, T. Shijuku, Y. Takano, L. Zulkifli, T. Yamazaki, A. Tominaga, S. Souma, K. Onai, M. Morishita, M. Ishiura, M. Hagemann, I. Suzuki, H. Maruyama, F. Arai, N. Uozumi, Comparative analysis of kdp and ktr mutants reveals distinct roles of the potassium transporters in the model cyanobacterium Synechocystis sp. strain PCC 6803. *J. Bacteriol.* **197**, 676–687 (2015).
125. J. Gibhardt, G. Hoffmann, A. Turdiev, M. Wang, V. T. Lee, F. M. Commichau, c-di-AMP assists osmoadaptation by regulating the *Listeria monocytogenes* potassium transporters KimA and KtrCD. *J. Biol. Chem.* **294**, 16020–16033 (2019).
126. Y. Bai, J. Yang, T. M. Zarrella, Y. Zhang, D. W. Metzger, G. Bai, Cyclic di-AMP impairs potassium uptake mediated by a cyclic di-AMP binding protein in Streptococcus pneumoniae. *J. Bacteriol.* **196**, 614–623 (2014).
127. A. M. Martorana, P. Sperandeo, A. Polissi, G. Dehò, Complex transcriptional organization regulates an Escherichia coli locus implicated in lipopolysaccharide biogenesis. *Res. Microbiol.* **162**, 470–482 (2011).
128. S. Shan, H. Min, T. Liu, D. Jiang, Z. Rao, Structural insight into dephosphorylation by trehalose 6-phosphate phosphatase (OtsB2) from Mycobacterium tuberculosis. *FASEB J.* **30**, 3989–3996 (2016).
129. R. Ruhal, R. Kataria, B. Choudhury, Trends in bacterial trehalose metabolism and significant nodes of metabolic pathway in the direction of trehalose accumulation. *J. Microbial. Biotechnol.* **6**, 493–502 (2013).
130. J. J. Hubloher, S. Zeidler, P. Lamosa, H. Santos, B. Averhoff, V. Müller, Trehalose-6-phosphate-mediated phenotypic change in Acinetobacter baumannii. *Environ. Microbiol.* **22**, 5156–5166 (2020).
131. S. Zeidler, J. Hubloher, K. Schabacker, P. Lamosa, H. Santos, V. Müller, Trehalose, a temperature- and salt-induced solute with implications in pathobiology of Acinetobacter baumannii. *Environ. Microbiol.* **19**, 5088–5099 (2017).

132. A. R. Strøm, I. Kaasen, Trehalose metabolism in Escherichia coli: Stress protection and stress regulation of gene expression. *Mol. Microbiol.* **8**, 205–210 (1993).
133. D. Szklarczyk, A. L. Gable, D. Lyon, A. Junge, S. Wyder, J. Huerta-Cepas, M. Simonovic, N. T. Doncheva, J. H. Morris, P. Bork, L. J. Jensen, C. von Mering, STRING v11: Protein-protein association networks with increased coverage, supporting functional discovery in genome-wide experimental datasets. *Nucleic Acids Res.* **47**, D607–D613 (2019).
134. S. A. E. Heider, N. Wolf, A. Hofemeier, P. Peters-Wendisch, V. F. Wendisch, Optimization of the IPP precursor supply for the production of lycopene, decaprenoxanthin and astaxanthin by Corynebacterium glutamicum. *Front. Bioeng. Biotechnol.* **2**, 28 (2014).
135. I. Chitrakar, S. F. Ahmed, A. T. Torelli, J. B. French, Structure of the E. coli agmatinase, SPEB. *PLOS ONE* **16**, e0248991 (2021).
136. L. Miller-Fleming, V. Olin-Sandoval, K. Campbell, M. Ralser, Remaining mysteries of molecular biology: The role of polyamines in the cell. *J. Mol. Biol.* **427**, 3389–3406 (2015).
137. M. Burnat, E. Flores, Inactivation of agmatinase expressed in vegetative cells alters arginine catabolism and prevents diazotrophic growth in the heterocyst-forming cyanobacterium Anabaena. *Microbiology* **3**, 777–792 (2014).
138. M. Zamakhaev, I. Tsyganov, L. Nesterova, A. Akhova, A. Grigorov, J. Bespyatykh, T. Azhikina, A. Tkachenko, M. Shumkov, *Mycolicibacterium smegmatis* possesses operational agmatinase but contains no detectable polyamines. *Int. J. Mycobacteriol.* **9**, 138–143 (2020).
139. C. Kotakis, E. Theodoropoulou, K. Tassis, C. Oustamanolakis, N. E. Ioannidis, K. Kotzabasis, Putrescine, a fast-acting switch for tolerance against osmotic stress. *J. Plant Physiol.* **171**, 48–51 (2014).
140. M. B. Szumanski, S. M. Boyle, Influence of cyclic AMP, agmatine, and a novel protein encoded by a flanking gene on speB (agmatine ureohydrolase) in Escherichia coli. *J. Bacteriol.* **174**, 758–764 (1992).
141. T. C. Marlovits, W. Haase, C. Herrmann, S. G. Aller, V. M. Unger, The membrane protein FeoB contains an intramolecular G protein essential for Fe(II) uptake in bacteria. *Proc. Natl. Acad. Sci. U.S.A.* **99**, 16243–16248 (2002).
142. B. Stevenson, E. E. Wyckoff, S. M. Payne, Vibrio cholerae FeoA, FeoB, and FeoC interact to form a complex. *J. Bacteriol.* **198**, 1160–1170 (2016).
143. M. L. Cartron, S. Maddocks, P. Gillingham, C. J. Craven, S. C. Andrews, Feo—Transport of ferrous iron into bacteria. *Biometals* **19**, 143–157 (2006).
144. B. M. Hopkinson, K. A. Barbeau, Iron transporters in marine prokaryotic genomes and metagenomes. *Environ. Microbiol.* **14**, 114–128 (2012).
145. A. E. Sestok, S. M. O'Sullivan, A. T. Smith, A general protocol for the expression and purification of the intact transmembrane transporter FeoB. *Biochim. Biophys. Acta Biomembr.* **1864**, 183973 (2022).
146. F. J. Cameron, M. V. Jones, C. Edwards, Effects of salinity on bacterial iron oxidation. *Curr. Microbiol.* **10**, 353–356 (1984).
147. C. Brochier, P. López-García, D. Moreira, Horizontal gene transfer and archaeal origin of deoxyhypusine synthase homologous genes in bacteria. *Gene* **330**, 169–176 (2004).
148. R. C. Moore, S. M. Boyle, Cyclic AMP inhibits and putrescine represses expression of the speA gene encoding biosynthetic arginine decarboxylase in Escherichia coli. *J. Bacteriol.* **173**, 3615–3621 (1991).
149. A. Muñoz-García, O. Mestanza, J. P. Isaza, I. Figueroa-Galvis, J. Vanegas, Influence of salinity on the degradation of xenobiotic compounds in rhizospheric mangrove soil. *Environ. Pollut.* **249**, 750–757 (2019).
150. G. Witt, Polycyclic aromatic hydrocarbons in water and sediment of the Baltic Sea. *Mar. Pollut. Bull.* **31**, 237–248 (1995).
151. J. Godocíková, M. Zámocký, M. Bucková, C. Obinger, B. Polek, Molecular diversity of katG genes in the soil bacteria Comamonas. *Arch. Microbiol.* **192**, 175–184 (2010).
152. M. Christensen-Dalsgaard, K. Gerdes, Two higBA loci in the Vibrio cholerae superintegron encode mRNA cleaving enzymes and can stabilize plasmids. *Mol. Microbiol.* **62**, 397–411 (2006).
153. B. Kedzierska, L.-Y. Lian, F. Hayes, Toxin-antitoxin regulation: bimodal interaction of YefM-YoeB with paired DNA palindromes exerts transcriptional autorepression. *Nucleic Acids Res.* **35**, 325–339 (2007).
154. R. H. Little, L. Grenga, G. Saalbach, A. M. Howat, S. Pfeilmeier, E. Trampari, J. G. Malone, Adaptive remodeling of the bacterial proteome by specific ribosomal modification regulates Pseudomonas infection and niche colonisation. *PLOS Genet.* **12**, e1005837 (2016).
155. L. Grenga, R. H. Little, G. Chandra, S. D. Woodcock, G. Saalbach, R. J. Morris, J. G. Malone, Control of mRNA translation by dynamic ribosome modification. *PLOS Genet.* **16**, e1008837 (2020).
156. J. Payandeh, R. Pfoh, E. F. Pai, The structure and regulation of magnesium selective ion channels. *Biochim. Biophys. Acta* **1828**, 2778–2792 (2013).
157. P. Seitz, H. Pezeshgi Modarres, S. Borgeaud, R. D. Bulushev, L. J. Steinbock, A. Radenovic, M. Dal Peraro, M. Blokesch, ComEA is essential for the transfer of external DNA into the periplasm in naturally transformable *Vibrio cholerae* cells. *PLOS Genet.* **10**, e1004066 (2014).
158. P. Seitz, M. Blokesch, Cues and regulatory pathways involved in natural competence and transformation in pathogenic and environmental Gram-negative bacteria. *FEMS Microbiol. Rev.* **37**, 336–363 (2013).
159. D. Springael, E. M. Top, Horizontal gene transfer and microbial adaptation to xenobiotics: New types of mobile genetic elements and lessons from ecological studies. *Trends Microbiol.* **12**, 53–58 (2004).
160. J. Felce, M. H. Saier Jr., Carbonic anhydrases fused to anion transporters of the SulP family: Evidence for a novel type of bicarbonate transporter. *J. Mol. Microbiol. Biotechnol.* **8**, 169–176 (2004).
161. G. D. Price, F. J. Woodger, M. R. Badger, S. M. Howitt, L. Tucker, Identification of a SulP-type bicarbonate transporter in marine cyanobacteria. *Proc. Natl. Acad. Sci. U.S.A.* **101**, 18228–18233 (2004).
162. A. S. Zolotarev, M. Unnikrishnan, B. E. Shmukler, J. S. Clark, D. H. Vandorpe, N. Grigorieff, E. J. Rubin, S. L. Alper, Increased sulfate uptake by E. coli overexpressing the SLC26-related SulP protein Rv1739c from Mycobacterium tuberculosis. *Comp. Biochem. Physiol. A Mol. Integr. Physiol.* **149**, 255–266 (2008).
163. S. L. Alper, A. K. Sharma, The SLC26 gene family of anion transporters and channels. *Mol. Aspects Med.* **34**, 494–515 (2013).
164. D. C. Webb, H. Rosenberg, G. B. Cox, Mutational analysis of the Escherichia coli phosphate-specific transport system, a member of the traffic ATPase (or ABC) family of membrane transporters. A role for proline residues in transmembrane helices. *J. Biol. Chem.* **267**, 24661–24668 (1992).
165. S. Suzuki, A. Ferjani, I. Suzuki, N. Murata, The SphS-SphR two component system is the exclusive sensor for the induction of gene expression in response to phosphate limitation in synechocystis. *J. Biol. Chem.* **279**, 13234–13240 (2004).
166. A. Västermark, M. H. Saier Jr., The involvement of transport proteins in transcriptional and metabolic regulation. *Curr. Opin. Microbiol.* **18**, 8–15 (2014).
167. S. B. Namugenyi, A. M. Aagesen, S. R. Elliott, A. D. Tischler, *Mycobacterium tuberculosis* PhoY proteins promote persister formation by mediating Pst/SenX3-RegX3 phosphate sensing. *MBio* **8**, e00494-17 (2017).
168. Y. Qi, Y. Kobayashi, F. M. Hulett, The pst operon of Bacillus subtilis has a phosphate-regulated promoter and is involved in phosphate transport but not in regulation of the pho regulon. *J. Bacteriol.* **179**, 2534–2539 (1997).
169. S. Burut-Archanai, J. J. Eaton-Rye, A. Incharoensakdi, Na^+-stimulated phosphate uptake system in Synechocystis sp. PCC 6803 with Pst1 as a main transporter. *BMC Microbiol.* **11**, 225 (2011).
170. K. S. Smith, J. G. Ferry, Prokaryotic carbonic anhydrases. *FEMS Microbiol. Rev.* **24**, 335–366 (2000).
171. S.-H. Fan, M. Matsuo, L. Huang, P. M. Tribelli, F. Götz, The MpsAB bicarbonate transporter is superior to carbonic anhydrase in biofilm-forming bacteria with limited CO2 diffusion. *Microbiol. Spectr.* **9**, e0030521 (2021).
172. S.-H. Fan, P. Ebner, S. Reichert, T. Hertlein, S. Zabel, A. K. Lankapalli, K. Nieselt, K. Ohlsen, F. Götz, MpsAB is important for Staphylococcus aureus virulence and growth at atmospheric CO_2 levels. *Nat. Commun.* **10**, 3627 (2019).
173. C. Merlin, M. Masters, S. McAteer, A. Coulson, Why is carbonic anhydrase essential to Escherichia coli? *J. Bacteriol.* **185**, 6415–6424 (2003).
174. R. P. Henry, Environmentally mediated carbonic anhydrase induction in the gills of euryhaline crustaceans. *J. Exp. Biol.* **204**, 991–1002 (2001).
175. S. C. Harris, S. Devendran, J. M. P. Alves, S. M. Mythen, P. B. Hylemon, J. M. Ridlon, Identification of a gene encoding a flavoprotein involved in bile acid metabolism by the human gut bacterium Clostridium scindens ATCC 35704. *Biochim. Biophys. Acta Mol. Cell Biol. Lipids.* **1863**, 276–283 (2018).
176. H. L. Doden, J. M. Ridlon, Microbial hydroxysteroid dehydrogenases: From alpha to omega. *Microorganisms* **9**, 469 (2021).
177. A. I. Prieto, S. B. Hernández, I. Cota, M. G. Pucciarelli, Y. Orlov, F. Ramos-Morales, F. García-del Portillo, J. Casadesús, Roles of the outer membrane protein AsmA of Salmonella enterica in the control of marRAB expression and invasion of epithelial cells. *J. Bacteriol.* **191**, 3615–3622 (2009).
178. R. Misra, Y. Miao, Molecular analysis of asmA, a locus identified as the suppressor of OmpF assembly mutants of Escherichia coli K-12. *Mol. Microbiol.* **16**, 779–788 (1995).
179. M. Deng, R. Misra, Examination of AsmA and its effect on the assembly of Escherichia coli outer membrane proteins. *Mol. Microbiol.* **21**, 605–612 (1996).
180. T. P. Levine, Remote homology searches identify bacterial homologues of eukaryotic lipid transfer proteins, including Chorein-N domains in TamB and AsmA and Mdm31p. *BMC Mol. Cell Biol.* **20**, 43 (2019).
181. A. Y. Golovina, M. M. Dzama, I. A. Osterman, P. V. Sergiev, M. V. Serebryakova, A. A. Bogdanov, O. A. Dontsova, The last rRNA methyltransferase of E. coli revealed: The

yhiR gene encodes adenine-N6 methyltransferase specific for modification of A2030 of 23S ribosomal RNA. *RNA* **18**, 1725–1734 (2012).
182. P. V. Sergiev, A. Y. Golovina, I. A. Osterman, M. V. Nesterchuk, O. V. Sergeeva, A. A. Chugunova, S. A. Evfratov, E. S. Andreianova, P. I. Pletnev, I. G. Laptev, K. S. Petriukov, T. I. Navalayeu, V. E. Koteliansky, A. A. Bogdanov, O. A. Dontsova, N6-methylated adenosine in RNA: From bacteria to humans. *J. Mol. Biol.* **428**, 2134–2145 (2016).
183. E. Kierzek, R. Kierzek, The thermodynamic stability of RNA duplexes and hairpins containing N6-alkyladenosines and 2-methylthio-N6-alkyladenosines. *Nucleic Acids Res.* **31**, 4472–4480 (2003).
184. Y. Wen, J. Feng, D. R. Scott, E. A. Marcus, G. Sachs, The pH-responsive regulon of HP0244 (FlgS), the cytoplasmic histidine kinase of *Helicobacter pylori*. *J. Bacteriol.* **191**, 449–460 (2009).
185. L. C. Metzger, N. Matthey, C. Stoudmann, E. J. Collas, M. Blokesch, Ecological implications of gene regulation by TfoX and TfoY among diverse Vibrio species. *Environ. Microbiol.* **21**, 2231–2247 (2019).
186. L. Attaiech, C. Granadel, J.-P. Claverys, B. Martin, RadC, a misleading name? *J. Bacteriol.* **190**, 5729–5732 (2008).
187. E. Katsiou, C. M. Nickel, A. F. Garcia, M. H. Tadros, Molecular analysis and identification of the radC gene from the phototrophic bacterium Rhodobacter capsulatus B10. *Microbiol. Res.* **154**, 233–239 (1999).
188. R. Colin, B. Ni, L. Laganenka, V. Sourjik, Multiple functions of flagellar motility and chemotaxis in bacterial physiology. *FEMS Microbiol. Rev.* **45**, fuab038 (2021).
189. E. E. Ganusova, L. T. Vo, T. Mukherjee, G. Alexandre, Multiple CheY proteins control surface-associated lifestyles of Azospirillum brasilense. *Front. Microbiol.* **12**, 664826 (2021).
190. A. Silale, S. M. Lea, B. C. Berks, The DNA transporter ComEC has metal-dependent nuclease activity that is important for natural transformation. *Mol. Microbiol.* **116**, 416–426 (2021).
191. R. Salzer, T. Kern, F. Joos, B. Averhoff, The Thermus thermophilus comEA/comEC operon is associated with DNA binding and regulation of the DNA translocator and type IV pili. *Environ. Microbiol.* **18**, 65–74 (2016).
192. S. Melville, L. Craig, Type IV pili in Gram-positive bacteria. *Microbiol. Mol. Biol. Rev.* **77**, 323–341 (2013).
193. L. M. Iyer, D. D. Leipe, E. V. Koonin, L. Aravind, Evolutionary history and higher order classification of AAA+ ATPases. *J. Struct. Biol.* **146**, 11–31 (2004).
194. C. M. Mageeney, B. Y. Lau, J. M. Wagner, C. M. Hudson, J. S. Schoeniger, R. Krishnakumar, K. P. Williams, New candidates for regulated gene integrity revealed through precise mapping of integrative genetic elements. *Nucleic Acids Res.* **48**, 4052–4065 (2020).
195. A. Trchounian, H. Kobayashi, Kup is the major K+ uptake system in Escherichia coli upon hyper-osmotic stress at a low pH. *FEBS Lett.* **447**, 144–148 (1999).
196. A. Rodriguez-Navarro, M. R. Blatt, C. L. Slayman, A potassium-proton symport in Neurospora crassa. *J. Gen. Physiol.* **87**, 649–674 (1986).
197. I. Tascón, J. S. Sousa, R. A. Corey, D. J. Mills, D. Griwatz, N. Aumüller, V. Mikusevic, P. J. Stansfeld, J. Vonck, I. Hänelt, Structural basis of proton-coupled potassium transport in the KUP family. *Nat. Commun.* **11**, 626 (2020).
198. J. Yu, B. Zhang, Y. Zhang, C.-Q. Xu, W. Zhuo, J. Ge, J. Li, N. Gao, Y. Li, M. Yang, A binding-block ion selective mechanism revealed by a Na/K selective channel. *Protein Cell* **9**, 629–639 (2018).

Acknowledgments: We would like to thank M. Buck for providing early access to the StratFreshDB MAGs that were included in this study. **Funding:** This study was supported by a grant from the Swedish Research Council VR to A.F.A. (grant no. 2021-05563). M.M. was supported by a grant from the Swedish research council for sustainable development, FORMAS (grant no. 2021-00546). A.F.A. was additionally supported by a grant from the Swedish research council for sustainable development, FORMAS (grant no. 2022-01515). Publication funding was provided by KTH Royal Institute of Technology. **Author contributions:** A.F.A. designed the study in consultation with all coauthors. K.T.J., M.M., A.F.A., Z.D., and L.F.D. analyzed the data. K.T.J. and M.M. wrote the first draft of the manuscript. All authors contributed to the interpretation of the data, revised the manuscript, and approved the final version before submission. **Competing interests:** The authors declare that they have no competing interests. **Data and materials availability:** All data needed to evaluate the conclusions in the paper are present in the paper and/or the Supplementary Materials. All the MAGs used in this study are made publicly available via their original publications as referenced in the text and Table 1. MAGs used in this study are also submitted to Figshare and are available via DOI: https://doi.org/10.6084/m9.figshare.22211917.v1. Data files describing intermediate data analysis steps, including the phylogenetic trees, and the code for in-house data transformation procedures are available at https://doi.org/10.17044/scilifelab.20732170.

Submitted 10 December 2022
Accepted 21 April 2023
Published 26 May 2023
10.1126/sciadv.adg2059
